# TMEM16F regulates pathologic α‐synuclein secretion and spread in cellular and mouse models of Parkinson's disease

**DOI:** 10.1111/acel.14387

**Published:** 2024-11-02

**Authors:** Stav Cohen‐Adiv, Fatima Amer‐Sarsour, Yevgeny Berdichevsky, Emily Boxer, Orly Goldstein, Mali Gana‐Weisz, Utkarsh Tripathi, Wote Amelo Rike, Gali Prag, Tanya Gurevich, Nir Giladi, Shani Stern, Avi Orr‐Urtreger, Dinorah Friedmann‐Morvinski, Avraham Ashkenazi

**Affiliations:** ^1^ The Department of Cell and Developmental Biology, Faculty of Medical and Health Sciences Tel Aviv University Tel Aviv Israel; ^2^ The School of Neurobiology, Biochemistry and Biophysics, the George S. Wise Faculty of Life Sciences Tel Aviv University Tel Aviv Israel; ^3^ Sagol School of Neuroscience Tel Aviv University Tel Aviv Israel; ^4^ Laboratory of Biomarkers and Genomics of Neurodegeneration, Neurological Institute Tel Aviv Sourasky Medical Center Tel Aviv Israel; ^5^ Sagol Department of Neurobiology, Faculty of Natural Sciences University of Haifa Haifa Israel; ^6^ School of Neurobiology, Biochemistry and Biophysics, the George S. Wise Faculty of Life Sciences Tel Aviv University Tel Aviv Israel; ^7^ Movement Disorders Division, Neurological Institute Tel Aviv Sourasky Medical Center Tel Aviv Israel; ^8^ Faculty of Medical and Health Sciences Tel Aviv University Tel Aviv Israel; ^9^ Brain Division Tel Aviv Sourasky Medical Center Tel Aviv Israel

**Keywords:** aggregate‐prone proteins, aging‐related diseases, extracellular secretion, lipid translocases, proteostasis

## Abstract

One of the main hallmarks of Parkinson's disease (PD) pathology is the spread of the aggregate‐prone protein α‐synuclein (α‐syn), which can be detected in the plasma and cerebrospinal fluid of patients as well as in the extracellular environment of neuronal cells. The secreted α‐syn can exhibit “prion‐like” behavior and transmission to naïve cells can promote conformational changes and pathology. The precise role of plasma membrane proteins in the pathologic process of α‐syn is yet to be fully resolved. The TMEM16 family of lipid scramblases and ion channels has been recently associated with cancer and infectious diseases but is less known for its role in aging‐related diseases. To elucidate the role of TMEM16F in α‐syn spread, we transduced neurons derived from TMEM16F knockout mice with a reporter system that enables the distinction between donor and recipient neurons of pathologic α‐synA53T. We found that the spread of α‐synA53T was reduced in neurons derived from TMEM16F‐knockout mice. These findings were recapitulated in vivo in a mouse model of PD, where attenuated α‐synA53T spread was observed when TMEM16F was ablated. Moreover, we identified a single nucleotide polymorphism in TMEM16F of Ashkenazi Jewish PD patients resulting in a missense Ala703Ser mutation with enhanced lipid scramblase activity. This mutation is associated with altered regulation of α‐synA53T extracellular secretion in cellular models of PD. Our study highlights TMEM16F as a novel regulator of α‐syn spread and as a potential therapeutic target in synucleinopathies.

## Graphical abstract

Parkinson's disease pathology is associated with cell‐to‐cell transfer of the aggregate‐prone protein α‐synuclein (α‐syn). Using patient genetics, murine, and cellular disease modeling, we reveal that the lipid scrambling activity of transmembrane protein 16F (TMEM16F) regulates pathologic α‐synA53T extracellular secretion and transfer.

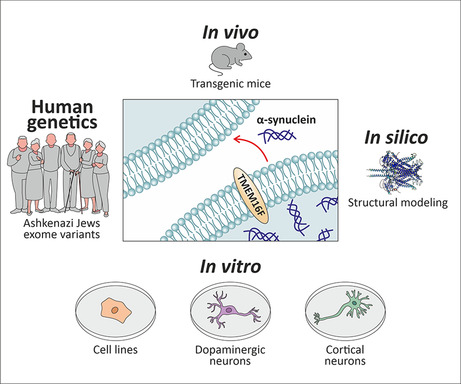

**Abbreviations**
AAV: adeno‐associated virus
AJ: Ashkenazi Jews
ANO6: anoctamin 6
DLB: dementia with Lewy bodies
EVs: Extracellular vesicles
iPSC: induced pluripotent stem cell
KD: knockdown
KO: knockout
LV: lentivirus
PD: Parkinson's disease
shRNA: short hairpin RNA
SNVs: single nucleotide variants
TMEM16F: transmembrane protein 16F
WT: wild type
α‐syn: α‐synuclein

## 1 INTRODUCTION

Aging is the greatest risk factor for developing Parkinson's disease (PD). An emerging area of research in PD and related synucleinopathies is the toxic spread of α‐synuclein (α‐syn) across the brain and the association of this spread with disease pathology (Luk et al., 2012; Sacino et al., 2014). The aggregate‐prone α‐syn is secreted to the extracellular environment, and studies in animal and cellular models have shown that this promotes toxicity via neuroinflammation and the trans‐synaptic spread of pathologic α‐syn to unaffected neurons (Emmanouilidou et al., 2010; Kim et al., 2019; Lee et al., 2014; Stuendl et al., 2021; Yan et al., 2024). A growing body of evidence suggests that neuronal spread of α‐syn may reflect a “prion‐like” phenomenon (Brundin & Melki, 2017).

α‐syn is a cytosolic protein that lacks a secretory signal peptide sequence. While it is still not fully understood by which mechanisms α‐syn can spread between cells, several unconventional secretion routes were proposed including non‐vesicular secretion or release of extracellular vesicles (Alvarez‐Erviti et al., 2011; Danzer et al., 2012; Wu et al., 2023). A fraction of α‐syn is identified both inside and outside extracellular vesicles and the presence of vesicles increases α‐syn aggregation (Danzer et al., 2012; Grey et al., 2015; Gustafsson et al., 2018).

One of the proteins that is linked to unconventional protein secretion, vesicle secretion, and membrane repair is ANO6/TMEM16F (Fujii et al., 2015; Han et al., 2019; Stewart et al., 2018; Whitlock & Hartzell, 2017; Wu et al., 2020). The anoctamin TMEM16 family of chloride channels are transiently activated by an increase in intracellular calcium and act as ion channels and lipid scramblases (Pedemonte & Galietta, 2014). ANO6/TMEM16F is a calcium‐dependent phospholipid scramblase that mediates phosphatidylserine (PS) cell‐surface exposure and regulates phospholipid distribution in the inner and outer leaflets of the plasma membrane (Suzuki et al., 2010). TMEM16F has several isoforms that are generated by alternative splicing (SV1–SV3, SV5, SV6), where the canonical SV1 is widely expressed in the brain. A human loss‐of‐function mutation in the *TMEM16F* gene causes Scott syndrome, a rare disorder of mild bleeding (Dachary‐Prigent et al., 1997; Suzuki et al., 2010).

On the basis of this knowledge, we set out to explore whether TMEM16F can trigger pathologic α‐syn secretion and neuron‐to‐neuron spread. For this purpose, we utilized an adeno‐associated virus (AAV)‐based system and preformed fibril (PFF) modeling of α‐syn spread. The results indicate a decreased pathologic α‐syn spread in TMEM16F‐depleted mouse neurons and brains, and the existence of a conserved TMEM16F human variant regulating extracellular secretion of pathologic α‐syn.

## 2 METHODS

### 2.1 Ethics approval

All participants provided an informed written consent before entering the study. All DNA samples were coded and tested anonymously. The Institutional and National Supreme Helsinki Committees for Genetics Studies (IRBs) approved the study protocols and informed consent. All mouse experiments were reviewed and approved by the Institutional Animal Care and Use Committee.

### 2.2 DNA extraction and genotyping of TMEM16F mice

The gene trap *TMEM16F*/*Ano6* KO mouse line C57BL/6‐*Ano6* ^*Gt(EUCJ0166e09)Hmgu*^/DgiJ was obtained from The Jackson Laboratory as previously described (Zhang et al., 2020). Adult mice were tagged, and an ear sample was taken in order to determine their genotype. DNA was extracted using the KAPA Express Extract kit (KK7101). Briefly, samples were incubated with extraction buffer, express enzyme, and DDW at 75°C for 15 min. The PCR reaction was initiated by adding 1 μL of DNA to a ready mix solution (#KK5620), DDW, and primers (sequence was provided by The Jackson Laboratory): common‐ AGATCTGTCTGTCTCTAACAACCA, WT‐ reverse GCTCGGGGTTCCAATCTCT, mutant reverse‐ GCTAGCACAACCCCTCACTC. PCR products were separated in a 2% agarose gel with ethidium bromide and analyzed by UV exposure. WT fragment size = 237 bp, mutant = 345 bp, heterozygous = 237 and 345 bp.

### 2.3 Viral vectors and cloning

We constructed adeno‐associated virus (AAV) vectors encoding the human synapsin‐1 promoter, designed to drive the expression of eGFP and Alanine 53 to Threonine mutant α‐synuclein and linked by a self‐cleaving P2A peptide (pAAV‐hSyn1‐eGFP‐P2A‐α‐synA53T‐HA), As the first stage, we generated an A53T mutation by Inverse PCR with primers hSynuclein A53T. FOR (5′–ACAACAGTGGCTGAGAAGACC‐ 3′) and hSynuclein A53T. REV (5′–CACACCATGCACCACTCCC – 3′) and with a lentiviral plasmid pCMV‐eGFP‐wild type α‐Synuclein‐HA tag used as a template. We then subcloned (applying Gibson assembly) the HA‐tagged A53T mutant α‐synuclein gene into the *Nco*I and *Hin*dIII sites of plasmid pAAV‐hSyn1‐eGFP‐P2A‐eGFPf‐WPRE‐HGHpA (a gift from Guoping Feng (Zhang et al., 2016), Addgene plasmid # 74513) with a lentiviral plasmid pCMV‐eGFP‐α‐Syn‐Puro A53T mut‐HA tag as a template, and forward and reverse primers, Syn‐*Nco*I‐gib.FOR (5′– TGAAACAAGCAGGGGATGTCGAAGAGAATCCCGGGCCAGCCATGGATGTATTCATGAAAGGACTTTCAAAGG–3′) and Syn‐*Hin*dIII‐gib.REV (5′– TCTTTCACAAATTTTGTAATCCAGAGGTTGATTATCGATAAGCTTTTAAGCGTAATCTGGAACATCGTATGGG–3′). Cloning produced the plasmid pAAV‐hSyn1‐eGFP‐P2A‐α‐syn A53T‐HA.

TMEM16F was targeted by shRNAs by employing the plasmid pAAV‐U6‐gRNA‐CBh‐mCherry (a gift from Jimok Kim (Li & Kim, 2017), Addgene plasmid # 91947). The sequence of the TMEM16F shRNA 1 is: 5′‐GGCTCACCCTCGGAGTATATA‐3′, TMEM16F shRNA 2 is: 5′‐CATCTACTCTGTGAAGTTCTTCATTTCCT‐3′, and the scrambled shRNA is: 5′‐GCACTACCAGAGCTAACTCAGATAGTACT‐3′ as previously described (Suzuki et al., 2010). The shRNAs were inserted as a primer duplex into the *Sac*I and *Xba*I sites of plasmid pAAV‐U6‐gRNA‐CBh‐mCherry.

As the first step towards construction of lentiviral vector containing human TMEM16F/ANO6 A703S–mCherry fusion protein under control of the CMV promoter, we used the forward and reverse primers, ANO6 A703S.FOR (5′– CATGGAAACTGACCACCCAG–3′) and ANO6 A703S.REV (5′– AGTCCACTCTTATTTCCAATATATTGTTCAC–3′) for site directed mutagenesis of plasmid pcDNA3.1‐hTMEM16F canonical variant 1 (kindly provided by Joo Hyun Nam). Next, used PCR with forward and reverse primers pCMV‐seq (5′‐TGGGCGGTAGGCGTGTACGG‐3′) and hANO6 *Psp*OMI.REV (5′‐GGGCCCGTTCTGATTTTGGCCGTAAATTGTTATC‐3′) to obtain the wild type and A703S mutant human TMEM16F genes from plasmid pcDNA3.1‐hTMEM16F.These were then subcloned into the *Nhe*I and *Psp*OMI sites of lentiviral vector pLVX‐mANO6‐mCherry‐C1 (a gift from Renzhi Han (Zhao et al., 2014), Addgene plasmid # 62554) in order to substitute the mouse TMEM16F gene for the human gene.

For lentiviral expression of A53T mutant human α‐synuclein linked to HA‐tag under the control of the CMV promoter, we performed site‐directed mutagenesis on plasmid pLL3.7‐α‐syn wt‐HA as a template (a gift from Ehud Gazit), followed by inverse PCR with forward hSynuclein A53T.FOR (5′‐ACAACAGTGGCTGAGAAGACC‐3′) and reverse primers hSynuclein A53T.REV (5′‐CACACCATGCACCACTCCC‐3′). Plasmid integrity was validated by DNA sequencing.

### 2.4 Isolation and culture of mouse primary cortical neurons

Primary mouse cortical neurons were isolated from wild‐type C57BL/6J or from homozygous C57BL/6N TMEM16F KO and wild‐type littermate embryos at E17. C57BL/6J wild type cerebral cortices were harvested and placed in ice‐cold HBSS under a dissection microscope. Embryos from heterozygous C57BL/6N TMEM16F female mouse were genotype by sampling the tail, and cerebral cortices were kept separated in medium (L15, 10% FBS, 1% P/S) until the genotype was verified. Cerebral cortices were then dissected and incubated in trypsin (T4049, Merck) and DNaseI (D4513, Merck) for 20 min followed by mechanical dissociation using sterile micropipette tips. Dissociated neurons were resuspended and cultured at 37°C in a humidified incubator with 5% CO_2_ and 95% O_2_ in poly‐D‐lysine coated 6‐well plates in neurobasal media (Thermofisher, 12349015) supplemented with 1% GlutaMAX™ Supplement (35050‐061, Thermofisher), 1% Sodium pyruvate (Thermofisher, 11360039), 2% B27 supplement (Thermofisher, 17504044), and 1% Penicillin–Streptomycin (03‐031‐1B, Sartorius). Spread was investigated by infecting neurons with AAV (PHP.S serotype) viral particles overnight at DIV 5 (1 μL of 9.4 × 10^11^ Genome Copies (GC)/ml virus in 100 μL media). Next, the culture medium was replaced by fresh medium and the neurons were cultured for a further 7 days without changing the medium. Drug treatment was assessed by supplementing the neuron culture medium with 100 nM Niclosamide (N0560000, Merck) on days 1 and 3 during the 7‐day spread period.

### 2.5 Generation and analysis of stable cell lines

HEK293T cells (ATCC CRL‐1573) were authenticated by STR profiling and were routinely tested for mycoplasma. The cells were grown in DMEM (01‐052‐1A, Sartorius) supplemented with 10% heat‐inactivated FBS (04‐007‐1A, Sartorius), 10000 units/mL penicillin, 10 mg/mL streptomycin (03‐031‐1B Sartorius), and 2 mM L‐glutamine (G7513, Merck) at 37°C with 5% CO_2_. Stable TMEM16F co‐expressing α‐synA53T cell lines were generated by infection of HEK293T cells with WT TMEM16F‐mCherry or mutant TMEM16F‐mCherry, or AAV encoding for the mCherry reporter. Samples of 8 × 10^6^ cells/ml were collected and positively selected for mCherry fluorescence (BD Aria III, Termo Fisher Scientific). After the cells were replated and allowed to recover, they were infected for a second time with α‐synA53T‐HA lentivirus. For the α‐syn extracellular secretion assay, infected cells were plated in 6‐well plates supplemented with 1 mL medium and cultured for 3 days. After this time, the media were collected, centrifuged (300 g) for 5 min, and filtered through a 0.2 μm syringe filter. Harvested cells and the filtered media were dissolved in Laemmli buffer containing 5% beta‐mercaptoethanol and analyzed by the Western blot analysis.

### 2.6 PFF‐induced cell‐to‐cell α‐synA53T transfer assay

The generation of preformed fibrils (PFF) from recombinant human α‐synA53T (S‐1002‐1, rPeptide) was previously described (Polinski et al., 2018). PFF were mixed with OptiMEM (11058021, Thermo Fisher Scientific) and Lipofectamine 2000 (11668019, Thermo Fisher Scientific). After 20 min, liposome preparations were added to stable HEK293T cell lines co‐expressing WT/mutant/no TMEM16F together with α‐synA53T‐HA in OptiMEM (final PFF concentration 1–5 μg/mL). Six hours later, the cells were washed and supplemented with full media. After 2 day, the media was changed to media‐free FBS for additional culture of 48 h. Then, conditioned media from the donor cells were collected and filtered through a 0.2 μm syringe filter. The filtered conditioned media was transferred to wild type acceptor HEK293T cells for 48 h incubation. The analysis of α‐synA53T‐HA transfer to acceptor cells was performed by immunostaining for α‐synA53T‐HA and by staining with the PROTEOSTAT® Aggresome detection kit (ENZ‐51035, Enzo Life Sciences) according to the manufacturer's protocol to detect aggregates (Ishtayeh et al., 2023).

### 2.7 Extracellular vesicles isolation

Stable HEK293T cell lines co‐expressing WT/mutant/no TMEM16F and α‐synA53T were plated on 10 cm plate. In some experiments, Stable HEK293T cell lines were treated with 5 μg/mL of α‐synA53T PFFs. When cells adhered, the media was changed to media‐free FBS for 48 h. Then, media was collected and centrifuged for 15 min (3000 ***g***). The supernatant was separated from the pellet, and adjusted to exoEaesy Maxi Kit (76064 Qiagen) protocol as previously described (Enderle et al., 2015). Purified isolated extracellular vesicles (EVs) were diluted in Laemmli buffer containing 5% beta‐mercaptoethanol and analyzed by Western blot.

### 2.8 Antibodies

The following antibodies were used in this study: mouse anti‐HA (Biolegend, 901501); rabbit anti‐HA (Cell signaling, 3724); mouse anti‐mCherry (Abcam, 125096); mouse anti‐α‐synuclein (BD Bioscience, 610787); mouse anti‐α‐synuclein (Santa Cruz, sc‐12767); rabbit anti‐α‐synuclein (phospho S129) (Abcam, ab51253); rabbit anti GFP (Abcam, ab6556); rabbit anti GFP (pAb) (Abcam 290); rabbit anti‐LC3 (Abcam, 192890); rabbit anti‐P62 (Abcam, 109012); mouse anti‐p53 (Calbiochem, OP03); mouse anti‐ubiquitin (P4D1) (Cell Signaling, 3936); rabbit anti‐phospho‐p70S6 Kinase (Thr389) (Cell Signaling, 9205); rabbit anti‐p70S6 Kinase (Cell Signaling, 2708); rabbit anti‐Actin (A2066, Merck); rabbit anti‐GAPDH (Abcam, 181602); mouse anti GAPDH (Abcam ab8245); mouse anti GM130 (BD 610823); mouse anti calnexin (Santa Cruz, sc‐23954); rabbit anti CD9 (Abcam, ab236630); mouse anti CD63 (Santa Cruz, sc‐5275); rabbit anti NFH (Merck, N4142); rabbit anti β‐Tubulin III (Merck T2200); Alexa Fluor® 555 (Abcam, 150078) conjugated goat anti‐rabbit secondary antibody; Alexa Fluor® 647 (Abcam, 150115) conjugated goat anti‐mouse secondary antibody; Alexa Fluor® 647 (Abcam, 150079) conjugated goat anti‐rabbit secondary antibody; Goat Anti‐Mouse IgG H&L (HRP) (Abcam, 6789); Goat Anti‐Rabbit IgG H&L (HRP) (Abcam, 6721).

### 2.9 Immunofluorescence staining and imaging

Neurons cultured on coverslips were washed and fixed in 4% paraformaldehyde (PFA) for 10 min. The cells were then permeabilized for 10 min in 0.1% Triton X‐100 and blocked in 2% BSA for 1 h. Primary antibodies (1:150) were added and incubated overnight at 4°C followed by incubation with secondary antibodies (1:500) for 2 h at room temperature. HEK293T cells were cultured on coverslips, washed, and then fixed in 4% PFA for 10 min. Nuclear staining was detected by DRAQ5 (ThermoFisher, 65‐0880‐92). A Zeiss 710 confocal microscope with a 63X oil‐immersion lens was used for confocal imaging.

### 2.10 Phospholipid scramblase activity

Primary neurons were cultured on coverslips in a 6‐well plate. In DIV 5 were washed with PBS, and incubated with a mixture of 250 nM ionomycin (I0634, Merck), and 1:100 annexin V‐FITC in calcium rich buffer (14085, Abcam) for 10 min at room temperature. The cells were removed and washed twice with calcium rich buffer to avoid release of the annexin V‐FITC, followed by FA fixation and immunofluorescence imaging. The effect of Niclosamide was examined by incubation with the drug for 1 h at 37°C prior to annexin V‐FITC exposure. Annexin V binding was evaluated by measuring the ratio of FITC intensity to that of NFH, as a general neuronal morphology marker. The mCherry positive TMEM16F knockdown or overexpressed HEK293T cells were trypsinized and 10^6^ cells/mL were collected and centrifuged at 400 ***g***. The medium was discarded, and the pellet was resuspended in 10 μM ionomycin, annexin V‐FITC (1:100) in calcium rich buffer mixture and incubated for 5 min on an orbital shaker. Tubes were placed on ice and the FITC fluorescence of the mCherry expressing cells was analyzed by flow cytometry (Cytoflex4L, Beckman Coulter).

### 2.11 Analysis of α‐synuclein spread in neurons

Neurons transduced with AAV encoding for eGFP‐P2A‐α‐synA53T‐HA were analyzed by immunofluorescence. Cell‐to‐cell spread of α‐synA53T‐HA was detected by measuring the GFP and HA intensity in segmented cell bodies using a custom‐made ImageJ macro in Fiji. In brief, cell bodies were detected by the NFH staining, either by segmentation using the “adaptive threshold” plug‐in, or by manually outlining them using the “oval” tool. The intensity of both eGFP and HA were then measured in these cell bodies. Cells were considered transmitted (recipient neurons) if the eGFP signal was absent (threshold determined by the fluorescence of non‐transduced neurons), and the HA intensity was above background (secondary antibody control only). Transduced donor neurons were determined as the population that is positive to both eGFP and HA from the total NFH positive neurons (eGFP^+^, HA^+^/NFH^+^). Acceptor neurons were assessed by the absence of eGFP and the presence of HA from the non‐transduced population (eGFP^−^, HA^+^/NFH^+^, HA^−^). Transduction efficiency was 60% consistent between WT and TMEM16F KO neurons.

### 2.12 iPSC‐derived dopaminergic neurons differentiation and treatment

Dopaminergic neurons were differentiated from human iPSC lines using an established protocol as we have previously described (Tripathi et al., 2024). The iPSCs were cultured to ~80% confluency before dissociation with Accutase (AT104, Innovative Cell Technology), and replated on Matrigel‐coated plates at a density of 900,000 cells per well in mTesR Plus medium (Stem Cell Technologies). On day 0 (the start day of differentiation), the medium was switched to KSR medium containing DMEM F‐12 with Glutamax, 15% KO‐SR, 1% NEAA, 0.1 mM β‐mercaptoethanol. The KSR medium was supplemented with 10 μM SB431542. From day 5 to day 10, the KSR medium was gradually replaced with N2 medium (DMEM F‐12 with Glutamax, 1% N2 supplement) with the following composition: 75% KSR: 25% N2 for days 5–6, 50% KSR: 50% N2 for days 7–8, and 25% KSR: 75% N2 for days 9–10. During this period, additional molecules were supplied: 100 nM LDN‐193189 from days 0 to 12, 2 μM purmorphamine and 0.25 μM SAG from days 1 to 6, and 100 ng/mL FGF8b from days 1 to 6. On day 11, the medium was replaced with B27 medium containing Neurobasal medium, 2% B27 supplement, 1% Glutamax, 10 ng/mL BDNF, 10 ng/mL GDNF, 1 ng/mL TGFβ3, 0.2 mM ascorbic acid, and 0.1 mM cAMP. 3 μM CHIR99021 was added from days 3 to 12. Neurons were dissociated between days 20 and 25 and replated on Matrigel‐coated coverslips. The neurons were allowed to mature for one more week in B27 medium before switching to BrainPhys medium (05790, Stem Cell Technologies). Following the second dissociation, neurons were replated onto 48 coverslips. Treatment with EVs was initiated after the second dissociation (around day 24). Dopaminergic neurons received EVs (2 μL) purified from conditioned media of α‐synA53T PFF‐treated stable HEK293T cell lines co‐expressing WT/mutant/no TMEM16F and α‐synA53T. Half of the media in each well was changed every other day, and the new media was supplemented with the appropriate treatment. One week after the second dissociation, coverslips were maintained in BP27 medium and were fixed between days 30 and 31 for subsequent immunostaining to analyze neurite morphology by Zeiss Axiovert fluorescence microscope.

### 2.13 Western blot analysis

Cells were washed with PBS and harvested in Laemmli buffer containing 5% beta‐mercaptoethanol. Protein samples were boiled for 5 min at 95°C, separated by SDS–PAGE, transferred onto PVDF membranes, subjected to the Western blot analysis. Membranes were blocked in freshly prepared TBS containing 5% nonfat dry milk with 0.05% Tween‐20 (TTBS) for 60 min at room temperature with constant agitation. Primary antibodies were diluted (1:500–1:2000) in fresh blocking solution and were incubated with the membranes overnight at 4°C. Secondary antibodies were diluted in TTBS (1:10,000) and incubated with membranes for 1 h at room temperature. Membranes were visualized using the ECL‐enhanced chemiluminescence reagent (CYANAGEN). Protein levels in each sample were evaluated by normalization to the housekeeping GAPDH. The bands were quantified using ImageJ software.

### 2.14 mRNA analysis by qRT‐PCR

RNA was purified with the Bio‐Tri RNA reagent (009010233100, Bio‐Lab) according to the manufacturer's protocol. The concentration and quality of the RNA were measured by NanoDrop spectrophotometer (Thermo Fisher). The cDNA samples were prepared with a cDNA kit (95047‐100‐2, Quantabio) as described in the kit protocol. Reaction solutions were prepared for each set of primers including the genes TMEM16F/GAPDH, and each biological repeat was assessed in triplicate. The reaction volume contained Fast SYBER green master mix (95073‐250, Quantabio), forward and reverse primers, ultrapure water, and the cDNA template. To calculate relative RNA expression, the mean GAPDH primers were included as an endogenous control. The data were analyzed using the ΔΔCT method. The sequence of the primers used were as follows:

**Table:** 

Gene name	Forward	Reverse
TMEM16F	CTCCGGGAGACATGCAGATG	GTCAAAGTTTTCCAGCACCAGAC
GAPDH	GACCACTTTGTCAAGCTCATTTC	CTCTCTTCCTCTTGTGCTCTTG

### 2.15 In vivo studies in mice and brain immunohistochemistry

Six‐week old C57BL/6 female mice were injected intravenously (*i.v*.) with AAV‐PHP.eB (10 μL of 1.3–2.5 × 10^13^ GC/mL virus +90 μL PBS) encoding for the mCherry reporter and for either a scrambled control, or for TMEM16F shRNA1 or TMEM16F shRNA2. Two weeks later, the mice were injected stereotaxically with 1 μL of AAV eGFP‐P2A‐α‐synA53T‐HA (stock of 4 × 10^13^ GC/mL) into the cortex (coordinates from bregma: AP = 0.8, ML = −1.0, DV = −1.0). For this purpose, the mice were anaesthetized with isoflurane (2%) and placed in a Kopf Sterotaxic Alignment System and injected as described previously (Magod et al., 2021). Six weeks after the first *i.v* injection, all the mice were perfused transcardially with cold PBS followed by 4% PFA in PBS. The brains were harvested, fixed in PFA overnight, and transferred to 30% sucrose in PBS. Coronal sections (40 μm) were cut using a HM450 Microtome (ThermoFischer Scientific) and floating sections were then washed, placed on coverslips, and outlined by hydrophobic pen. The sections were permeabilized in 1.25% TritonX‐100 for 15 min at room temperature and then blocked with 0.25% TritonX‐100, 3% donor goat serum (DGS) (04‐009‐1A, Sartorius) in PBS for 1 h. Primary antibody was diluted in dilution buffer: 0.25% TritonX‐100, 0.3% DGS in PBS at a ratio of 1:150 and incubated with the samples overnight at 4°C. Secondary antibody was diluted in dilution buffer in 1:500 and incubated for 2 h before the samples were mounted and imaged using Zeiss 710 confocal microscope. Confocal imaging used a 20X air‐immersion lens, with the images collected as tiles followed by stitching the mosaic image. The coronal sections mosaics were obtained by an operator who was blinded to the experimental conditions. Threshold setting for eGFP and HA staining with secondary antibody only were determined on control PBS injected brain slices.

### 2.16 Proteomics analysis

Mouse primary cortical neurons (treated with control scrambled shRNA or TMEM16F shRNA2, *n* = 4) were harvested in Laemmli buffer supplemented with beta‐mercaptoethanol. Samples were boiled for 5 min at 95°C, and equivalent amount of whole protein samples were resolved and digested in‐gel. The resulting tryptic peptides were analyzed by LC‐MSMS using a Q Exactive (Thermo Scientific). Raw files were processed using the DiANN identification and quantification against mouse proteome from the Uniprot database. Differentially expressed genes (DEGs) with *p* value cutoff *p* < 0.05 between the groups (control shRNA vs. TMEM16F shRNA) were identified. Function enrichment of the identified DEGs was analyzed using DAVID (v2022q3 release) (Sherman et al., 2022).

### 2.17 Study cohort of PD patients

The PD cohort comprises 1200 unrelated patients of full Ashkenazi‐Jewish (AJ) origin, who were consecutively recruited between 2005 and 2016 (age at motor symptoms onset 60.56 ± 10.96). All patients were examined at the Movement Disorder Center at the Tel‐Aviv Sourasky Medical Center as previously described (Goldstein et al., 2019). Among the 1200 patients, 34% harbor a *GBA* mutation, a *LRRK2*‐mutation (G2019S), or both (Table S2). The other 787 patients (65.6%) do not carry any of these mutations, or the *SMPD1*‐L302P mutation (noncarriers, NC‐PD). Principal component analysis (PCA) and identity‐by‐descent analysis were performed on 591 of the 1200 AJ‐PDs using Affymetrix Genome‐Wide Human SNP Array 6.0 data (the Tel Aviv PD SNP6.0 array data was described in (Vacic et al., 2014)) to confirm Ashkenazi‐Jewish ethnicity and to rule out hidden relatedness.

### 2.18 Whole‐genome‐sequencing analysis

Whole‐genome‐sequencing (WGS) was carried out on 250 patients of Ashkenazi origin (AJ) affected with Parkinson's disease (PD, *n* = 231) or Dementia with Lewy Bodies (DLB, *n* = 19) as previously described (Goldstein et al., 2021). Briefly, sequencing was conducted by DNBseq technology, at BGI, China, with paired‐end reads (each of 100 bp length), and at 30×‐depth‐coverage (*n* = 221) or 10×‐depth‐coverage (*n* = 29). Paired‐end reads were aligned to the human reference genome GRCh38/hg38 build using the BWA tool (Li & Durbin, 2009), and the Genome Analysis Toolkit (GATK) was applied to the alignment data of each sample for variant calling (McKenna et al., 2010). Variants were extracted from a 234 Kb interval flanking *TMEM16F* (hg38: Chr12:45,211,095‐45,445,404) using SNP & Variation Suite V.8.9.0 (Golden Helix, Inc.). Variants with a read depth below five, or genotype quality below 30 were filtered out, and the rest were annotated and evaluated for functional effect using the prediction tool CADD (Combined Annotation Dependent Depletion, version 1.6) (Kircher et al., 2014; Rentzsch et al., 2019, 2021). Amino acid conservation and evolutionary constraints were estimated by Aminode (Chang et al., 2018).

### 2.19 Patient genotyping and statistical analysis

*TMEM16F*‐p.Ala703Ser (c.2107G > T, rs202121654) was genotyped on the complete cohort of 1200 AJ‐PD patients (Thermo Fisher Scientific fluorescent TaqMan® assays: C_189946725_10; StepOnePlus RT‐PCR system (Applied Biosystems)). Odds ratios (ORs) and 95% confidential intervals (CI) were determined by comparing allele counts in the PD cohort to those in gnomAD, the largest genome aggregated database available to‐date for Ashkenazi Jewish (AJ) controls (Karczewski et al., 2020). We used the gnomAD‐AJ‐non‐neuro cases (individuals who were diagnosed with a non‐neurological condition in a neurological case/control study). As the allele frequencies were not significantly different between gnomAD‐AJ‐non‐neuro cases version 2.1.1 and version 3.1.2 (*p* = 0.6048), we combined the allele counts from version 2.1.1 and version 3.1.2‐non‐v2, to avoid duplicates. ORs were calculated with an online calculator (https://www.medcalc.org). Effect on age‐at‐disease‐onset was estimated by linear regression, with sex and *ANO6*‐p.Ala703Ser carrier status as covariates (SPSS software V25 SPSS Inc., Chicago, IL).

### 2.20 Structural modelling and analysis

We have employed alphafold2 (Jumper et al., 2021) and Phyre2 (Kelley et al., 2015) to model dimeric TMEM16 of the human and the mouse protein sequences with and without Ca^2+^. The qualities of the models were assessed by inspection of the structures and the obtained predicted Local Distance Difference Test (pLDDT) and the Predicted Aligned Error (PAE) using ChimeraX. We have used Pymol for further analysis of mutations including Adaptive Poisson‐Boltzmann Solver (APBS) to calculate and render the electrostatics potential surface and for figure preparation.

## 3 RESULTS

### 3.1 TMEM16F contributes to neuron‐to‐neuron spread of pathologic α‐synuclein

**FIGURE 1:**
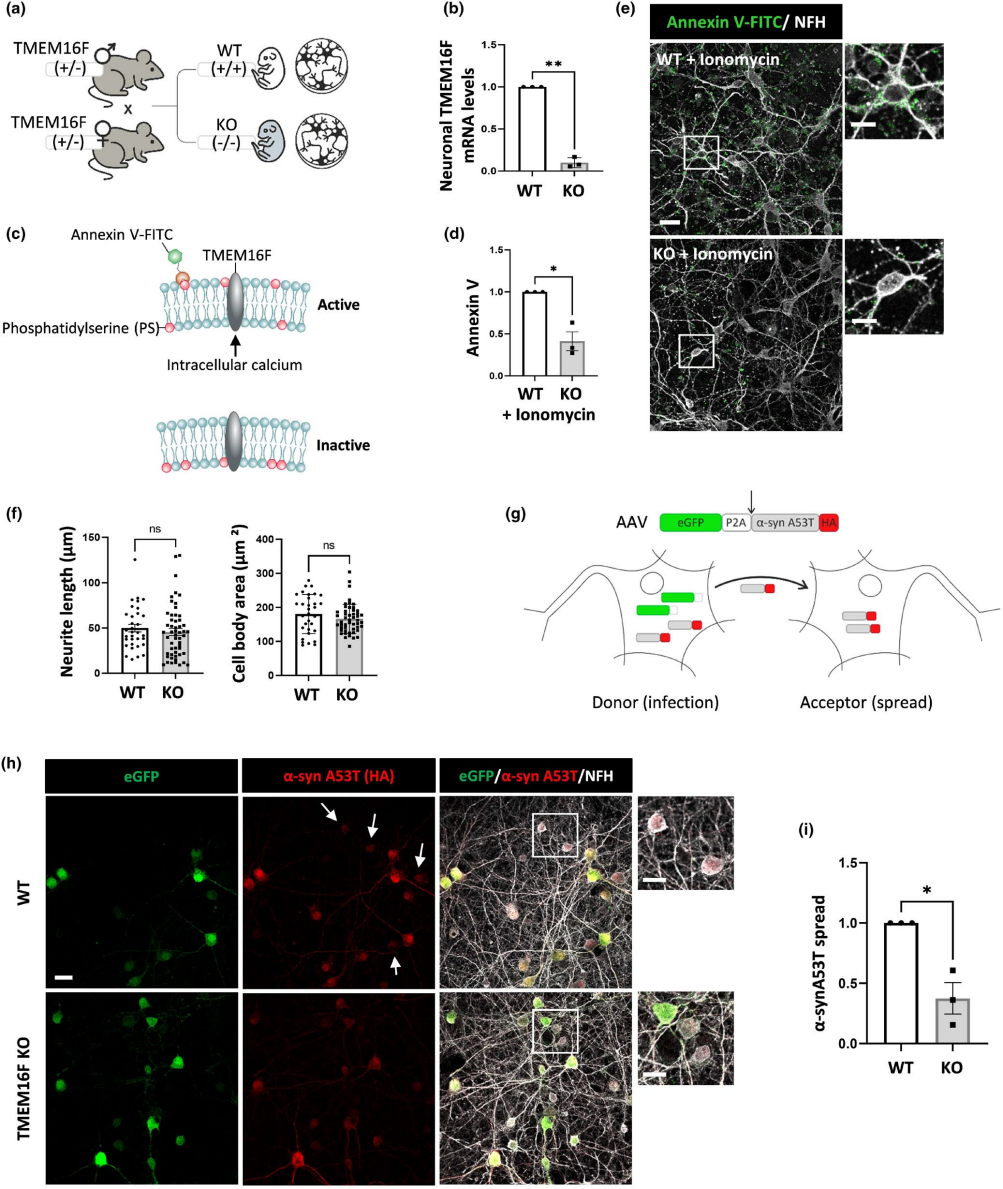
Reduced α‐synucleinA53T spread in primary neurons derived from TMEM16F knockout mice. (a) Heterozygous mice for the TMEM16F knockout (KO) allele (C57BL/6‐*Ano6* ^*Gt(EUCJ0166e09)Hmgu*^/DgiJ mice) were crossed and primary cortical neurons were cultured from brains of E17 WT and KO embryos. (b) qRT‐PCR quantification of TMEM16F mRNA levels relative to GAPDH in the primary neurons (*n* = 3 independent cultures). (c) Representation of TMEM16F activity assay: Intracellular calcium‐dependent phospholipid scrambling of TMEM16F increases transbilayer movement of phosphatidylserine (PS) from the inner to the out leaflet, which can be detected by recombinant FITC‐Annexin V binding. (d, e) Analysis of extracellular PS exposure in ionomycin‐treated (250 nM for 10 min) TMEM16F WT and KO primary neurons by FITC‐Annexin V binding. Confocal images are presented for FITC fluorescence (colored green) and neuronal morphology marker NFH (colored gray). Scale bar 20 μm (insets scale 10 μm). FITC intensity in different image fields was obtained and normalized to control WT neurons. *n* = 3 independent cultures. (f) Analysis of neurite length and cell body area in the NFH‐stained TMEM16F WT and KO neurons. Results are average of the morphological parameters in different image fields in *n* = 3 independent cultures. (g) Diagram of the AAV system encoding eGFP‐P2A‐α‐synA53T‐HA used to investigate neuronal α‐syn spread. (h, i) TMEM16F WT and KO neurons were transduced with the AAV system and spread was analyzed after one‐week post infection. (h) NFH staining (colored gray), eGFP (colored green), α‐synA53T‐HA (HA staining, colored red). Images are shown for the transduced donor neurons and acceptor neurons (scale bar 20 μm). Acceptor neurons are marked with arrows and are shown in insets (inset scale bar 10 μm). (i) Approximately 200 neuronal cell bodies were quantified per experiment. The results of spread were normalized to control neurons and calculated as described in the Methods. *n* = 3 independent cultures. 2‐tailed *t*‐test *p < 0.05, **p < 0.01.

We generated a TMEM16F knockout (KO) phenotype in neurons by crossing male and female heterozygous C57BL/6‐*Ano6* ^*Gt(EUCJ0166e09)Hmgu*^/DgiJ mice with the KO allele (Figure 1a). Primary cortical neurons derived from TMEM16F KO mouse embryos have no detectable TMEM16F mRNA and bind less recombinant Annexin V after ionomycin treatment than neurons of WT littermates, indicating lower levels of cell surface PS (Figure 1b–d). This suggests that KO neurons have lower phospholipid translocase activity in the plasma membrane although there were no overt morphological differences (average cell body size or neurite length) observed between the WT and KO neurons (Figure 1f).

We examined whether TMEM16F regulates the spread of α‐synuclein A53T (α‐synA53T) harboring the familial PD mutation A53T in the *SNCA* gene, a mutation that correlates with enhanced α‐syn binding to membranes (Jo et al., 2000). The α‐synA53T was selected as a trigger in order to enhance pathological events to be detected in a defined time‐scale. We modified a recently reported model of tau spread that can discriminate between transduced donor neurons and recipient neurons that receive tau through spread (Rauch et al., 2020) to measure the spread of α‐synA53T (Figure 1g). The AAV vector used encodes one mRNA of eGFP‐P2A‐α‐synA53T‐HA under the control of a synapsin (hSyn) promoter. Since ribosomes skip the glycine‐proline bond in P2A synthesis, this generates two proteins, namely eGFP‐P2A and α‐synA53T tagged with HA peptide (α‐synA53T‐HA). This system enabled us to discriminate between donor transduced neurons, which express eGFP and α‐synA53T‐HA, and recipient neurons, which include α‐synA53T‐HA (no eGFP) as a result of cell‐to‐cell α‐synA53T‐HA spread. Importantly, we designed this system to monitor early events of cell‐to‐cell spread. We examined the α‐synA53T‐HA spread 1 week after transduction of WT and TMEM16F KO neurons with the AAV by using an imaging algorithm to analyze the eGFP and HA staining intensity in 200 neuronal cell bodies for an individual experiment (Figure 1g). The results revealed that α‐synA53T‐HA spread was significantly lessened in TMEM16F KO neurons as compared to WT littermates (Figure 1g–i).

### 3.2 Effects of TMEM16F on protein degradation and pathologic α‐synuclein levels

In an attempt to examine whether other mechanisms are involved in TMEM16F‐regulated α‐synA53T spread, we analyzed the effects on α‐synA53T levels that correlate with α‐syn pathologic propensities. Notably, we did not detect any significant differences in the total levels of α‐synA53T between WT and KO neurons nor in aggregate‐prone protein degradation pathways, which suggests that the turnover rate of α‐synA53T is not affected by TMEM16F (Figure S1). Inhibition of proteasome‐mediated degradation resulted in the accumulation of ubiquitinated proteins. Therefore, we used cellular ubiquitination levels as a readout to measure effects on proteasome activity in TMEM16F KO neurons and specifically p53 levels that is tightly regulated by this process (Korolchuk et al., 2009). There were no differences in total cellular ubiquitination or in the levels of the proteasome substrate, p53 between TMEM16F WT and KO neurons (Figure S1a–c). Since α‐synA53T is also degraded by autophagy, we measured autophagosome load by analyzing the levels of lipidated LC3 (LC3‐II) on autophagosomal membranes relative to a loading control, such as actin (Ishtayeh et al., 2023; Webb et al., 2003). We observed a reduction in autophagosome load in the TMEM16F KO neurons, as indicated by a decrease in LC3‐II levels, although the levels of the autophagy substrate, p62 remained unaffected (Figure S1d,e). In addition, no significant difference was detected in the mTORC1 signaling to autophagy, measured by Thr389 phosphorylation of p70S6 kinase (Figure S1f). Overall, this indicates that the decrease in α‐synA53T spread in TMEM16 KO neurons was not due to perturbations in the total levels of the protein and subsequent degradation.

### 3.3 Targeting TMEM16F modulates pathologic α‐synuclein spread in vivo

Based on the in vitro results, we were interested to investigate whether TMEM16F loss‐of‐function diminishes the spread of α‐synA53T in the brain. The FDA‐approved drug Niclosamide inhibits several anoctamin members, including TMEM16F and TMEM16A (Cheng et al., 2023), and thus we considered this drug as potential therapeutic candidate for preventing α‐synA53T spread. Like the TMEM16F KO neurons, Niclosamide‐treated neurons exhibited lower Annexin V binding to extracellular PS than was observed in untreated neurons (Figure S2a). The nanomolar concentrations of the drug used had no apparent effects on neuronal morphology (Figure S2b). Strikingly, in contrast to TMEM16F KO neurons, α‐synA53T spread was increased in the Niclosamide‐treated neurons (Figure S2c), precluding the use of Niclosamide as an inhibitor of neuronal α‐syn spread.

**FIGURE 2:**
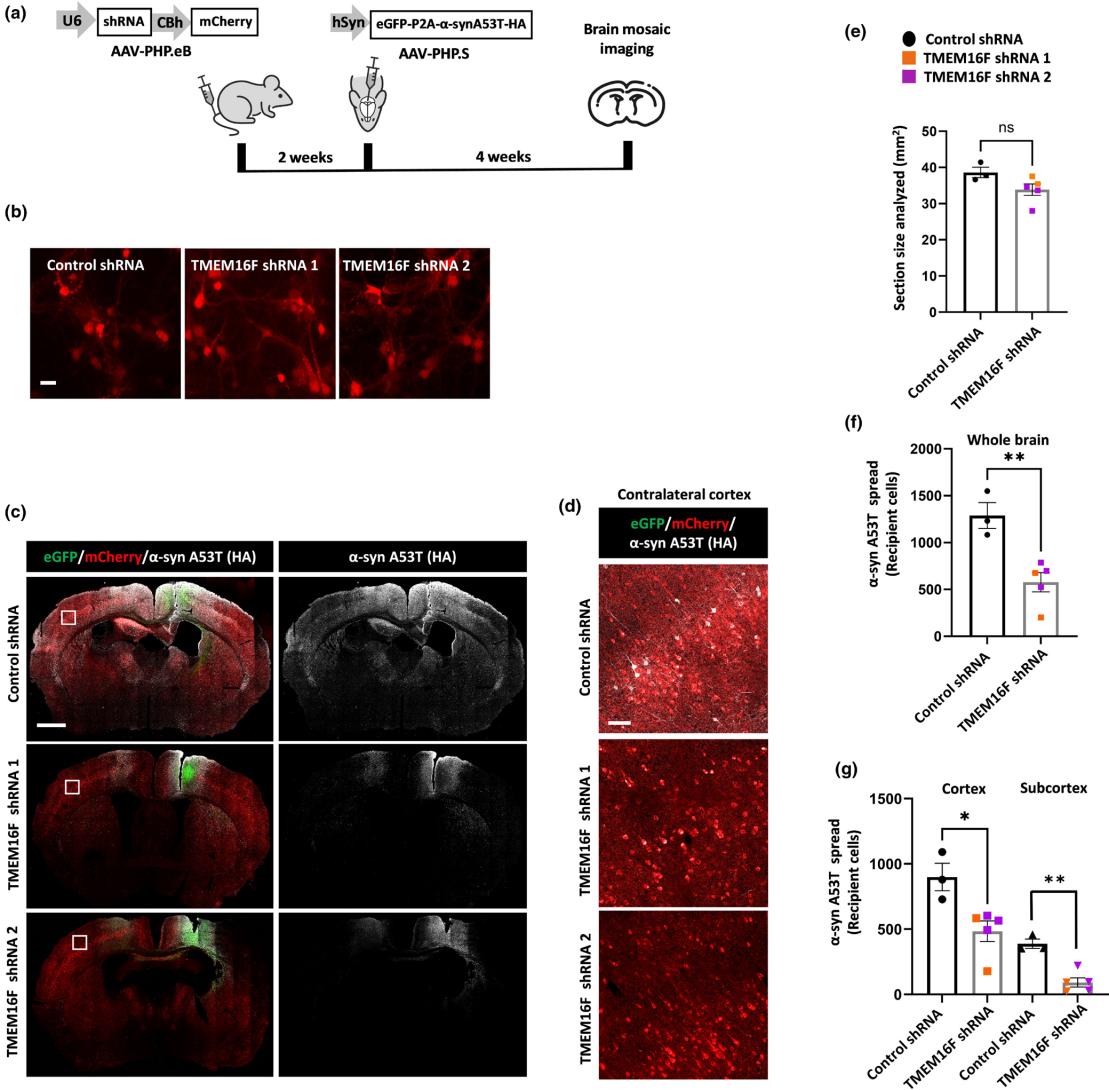
TMEM16F knockdown diminishes α‐synucleinA53T spread in vivo. (a) Diagram of the in vivo experimental design of two‐step AAV injections for silencing TMEM16F using two independent shRNAs followed by expression of the α‐synA53T spreading system. (b) Primary cortical neurons were transduced with AAV encoding for control scrambled shRNA or for one of two different TMEM16F shRNAs (TMEM16F targeting sh1 or sh2) as well as encoding for mCherry reporter. Images show robust expression of the shRNAs (mCherry, colored red). Scale bar 10 μm. (c) Representative mosaic of mice injected with AAV encoding for control scrambled shRNA, TMEM16F shRNA 1, or TMEM16F shRNA 2. Scale bar 1 mm. (d) Insets show the view of recipient neurons (mCherry^+^ colored red/ eGFP ^−^/α‐syn A53T‐HA^+^, colored gray) at the contralateral cerebral cortex. Scale bar 50 μm (e) Quantification of brain section sizes of mice used for analysis. (f) Whole brain (contralateral and ipsilateral side) quantification of mCherry^+^/ eGFP ^−^/ α‐syn A53T‐HA^+^ recipient cells in mice injected with scrambled shRNA or TMEM16F shRNAs. (g) Quantification of mCherry^+^/ eGFP ^−^/ α‐syn A53T‐HA^+^ recipient cells of whole brain divided to cortical and subcortical regions in mice injected with scrambled shRNA or TMEM16F shRNAs. *n* = 3–5 mice per group. 2‐tailed *t*‐test. ns, nonsignificant, *p < 0.05, **p < 0.01.

As an alternative approach, we genetically ablated endogenous TMEM16F expression in the brain. Since the TMEM16F KO phenotype is associated with partial perinatal lethality in mice (Zhang et al., 2020), we knocked down (KD) brain expression of TMEM16F by using two different shRNAs (Figure 2a). The AAVs (PHP.eB serotype (Chan et al., 2017)) co‐expressing the TMEM16F shRNAs and the mCherry reporter were intensely expressed in murine cortical neurons (Figure 2b) with a corresponding reduction in TMEM16F mRNA levels as compared to control scrambled shRNA expression (Figure S3a). Moreover, TMEM16F KD neurons transduced with the AAV eGFP‐P2A‐α‐synA53T‐HA have decreased pathologic phosphorylation of α‐syn at Serine 129 (α‐syn S129 (Anderson et al., 2006)) in recipient cells (Figure S3b,c).

AAVs encoding for one of the two different shRNAs targeting TMEM16F or the control scramble shRNA were administered via intravenous tail vein injection into 6‐week‐old WT female mice followed by stereotactic injections of the AAV eGFP‐P2A‐α‐synA53T‐HA (neuronal hSyn promoter, PHP.S serotype) into the cerebral cortex. Four weeks later, the mice were euthanized and α‐synA53T‐HA spread was detected by immunofluorescence analysis (Figure 2a,c). Expression of the AAV shRNA in the brain could be detected by imaging the mCherry reporter (Figure 2c,d). To quantify α‐synA53T‐HA spread, we counted the number of recipient cells in similarly sized WT and TMEM16F KD brain sections that were positive for α‐synA53T‐HA and mCherry but negative for eGFP (Figure 2d,e). The results revealed a considerable amount of α‐synA53T‐HA spread in the brains of mice injected with the scrambled shRNA, but the degree of spread was significantly reduced in the brains of TMEM16F KD mice (Figure 2f). This reduction was detected in the cortex (46% decrease in spread) and was more prominent in the subcortical structures (76% decrease in spread, Figure 2g).

Next, we assessed the proteome profile between control and TMEM16F KD neurons to reveal altered cellular pathways that could be involved in the spread of α‐synA53T. We were able to identify 675 differentially expressed genes (DEGs) between control and TMEM16F KD neurons (*p* < 0.05) (Table S1). Gene ontology (GO) and pathway enrichment analysis revealed processes related to membrane‐bounded organelle (synaptic vesicle transport, multivesicular body assembly, extracellular vesicle, and late endosome membrane), mitochondria (cellular respiration, electron transport, and oxidative phosphorylation), and calcium ion homeostasis (Figure S3d, Table S1). The proteomics results provide a resource for understanding the neuronal pathways affected by TMEM16F. In the following results below, we focus our analysis on selected pathways related to α‐synA53T spread in the context of human variants in TMEM16F.

### 3.4 Genetic variation occurs in the human *TMEM16F* gene

**FIGURE 3:**
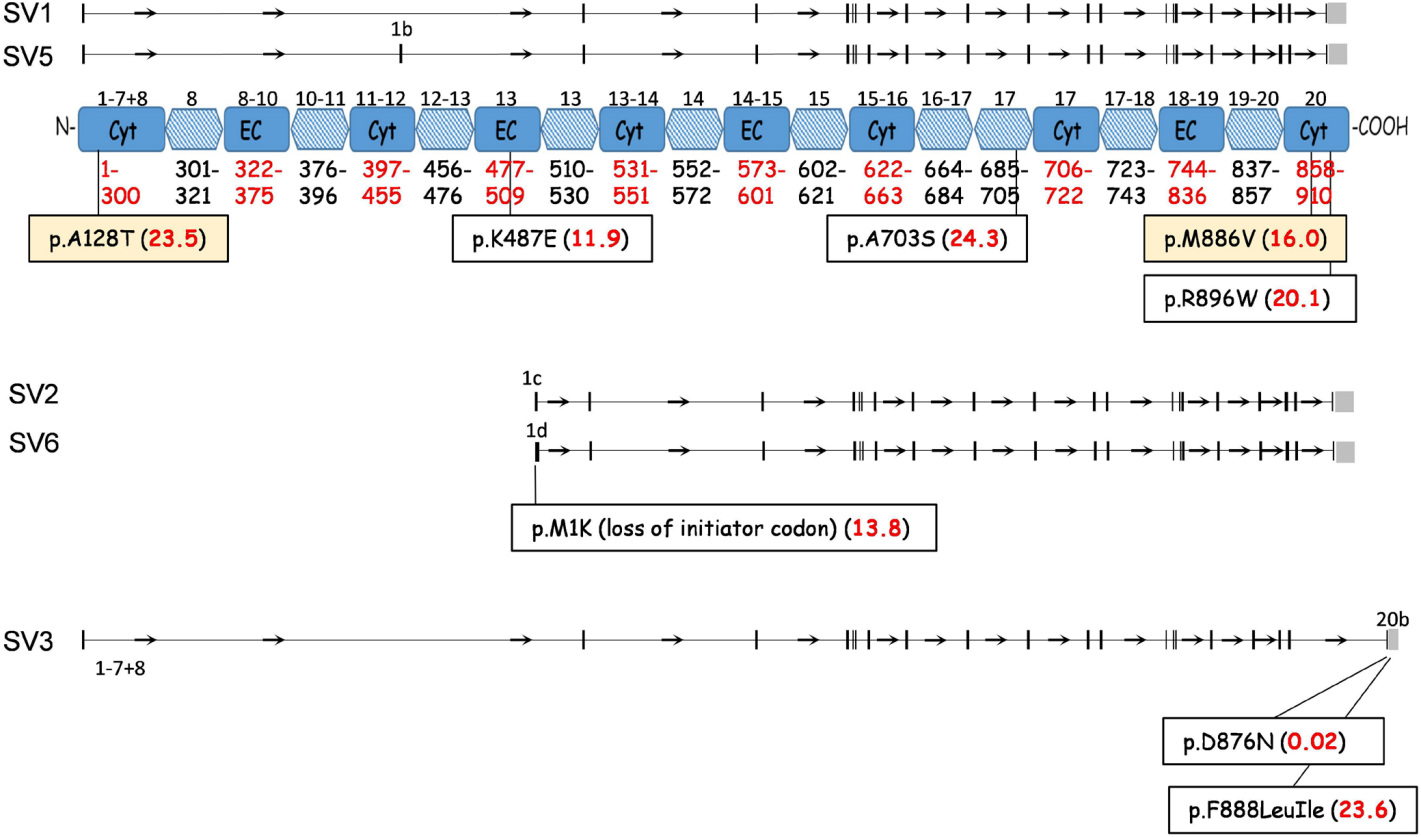
Variations in the TMEM16F gene observed in the Ashkenazi Jewish PD/ DLB patients. There are five TMEM16F splice variants (SV1, 2, 3, 5, 6) in humans. Eight single nucleotide variants (SNVs) were observed in whole‐genome‐sequencing of 250 Ashkenazi Jewish (AJ)‐PDs/DLBs. Light blue domains are predicted to be TMEM16F transmembrane domains. Dark blue domains are topological domains. Cyt, cytoplasmic; EC, extracellular. Numbers in parentheses are Phred‐CADD scores for functional prediction.

In order to understand how physiological perturbation in TMEM16F may affect pathologic α‐syn spread, we analyzed genetic variation in this gene. We identified eight coding single nucleotide variants (SNVs) in a cohort of 250 patients with Ashkenazi Jewish (AJ) origin and with PD or dementia with Lewy bodies (DLB) (Figure 3, Table S2). Three of the SNVs, namely p.Ala128Thr, p.Lys487Glu, and p.Ala703Ser, are shared among all *TMEM16F* isoforms (Figure 3, Figure S4). The other SNVs, p.Met886Val and p.Arg896Trp, which are located at the 3′ end, are common to all isoforms except isoform 3. The SNV that confers a probable loss of function effect, due to loss of initiator codon, p.Met1Lys, is unique to isoforms 2 and 6, while two SNVs, p.Asp876Asn and p.Phe888delinsLeuIle, which are located at the C‐terminus of the protein, are unique to isoform 3. In addition, among these SNVs, p.Ala128Thr and p.Met886Val are linked (Table S2, Figure S4).

The SNV with the highest functional prediction Phred‐CADD score of 24.3 is p.Ala703Ser, which is located in exon 17 of the canonical *TMEM16F* (Figure 3, Table S2). This variant is predominantly observed in the AJ population. The variant changes amino acid 703 from alanine to serine (A703S), which resides within a transmembrane domain in the predicted anoctamin calcium‐activated chloride channel domain (amino acids 287‐870), in a highly conserved amino acid within an evolutionary constrained region (Figure S5).

### 3.5 TMEM16F regulates extracellular secretion of pathologic α‐synuclein

**FIGURE 4:**
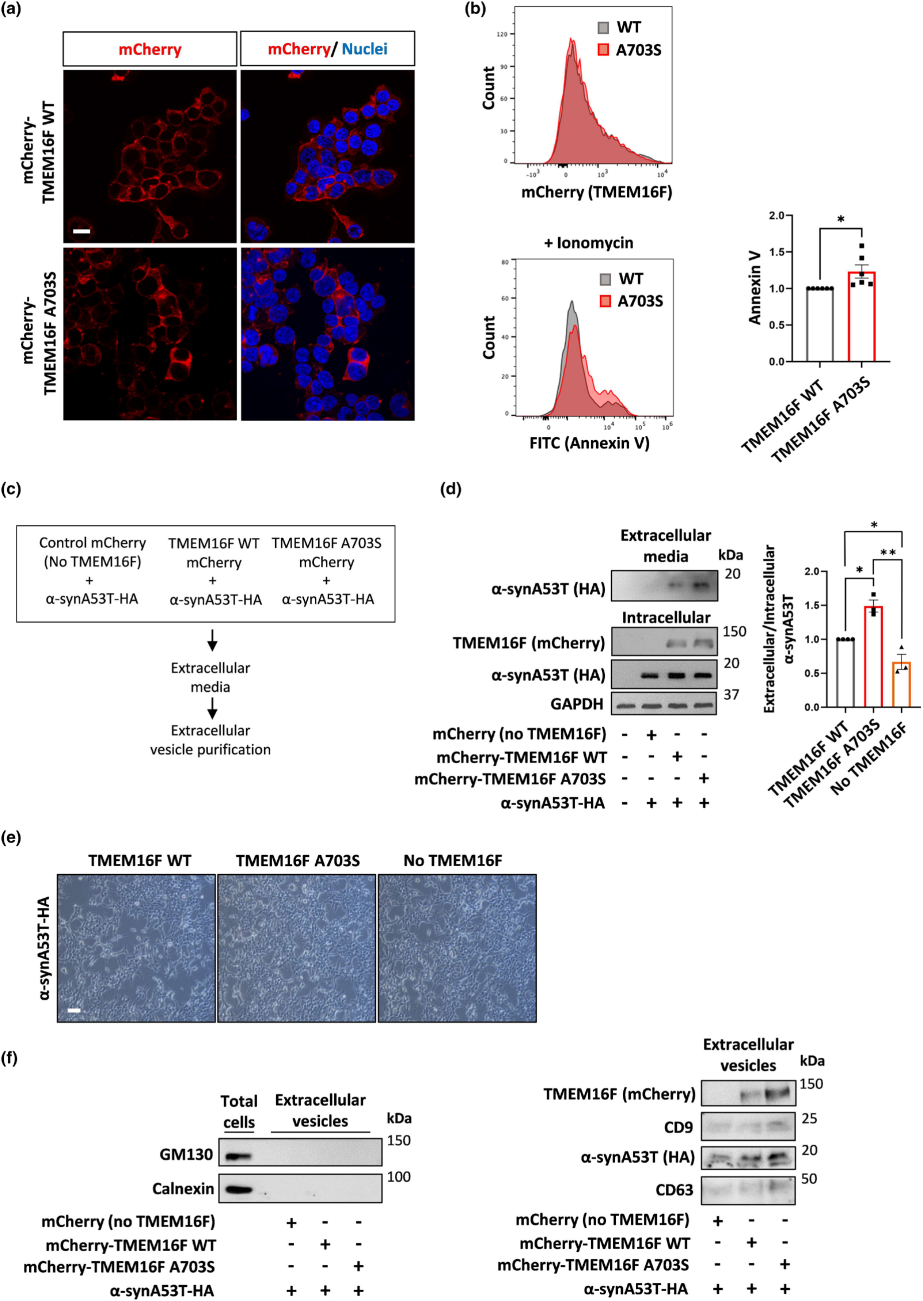
Functional perturbations in TMEM16F regulate the extracellular secretion of α‐synucleinA53T. (a) HEK293T cells were transduced with lentiviruses expressing mCherry‐TMEM16F WT or mCherry‐TMEM16F A703S and were analyzed for TMEM16F cellular expression by imaging. Scale bar 10 μm. (b) Flow cytometry analysis of Annexin V binding to extracellular PS in WT and A703S mutant TMEM16F stably‐expressing cells that were pre‐treated with ionomycin. Representative histograms are presented for Annexin V binding (FITC signal) in the TMEM16F‐expressing cells (mCherry signal). Results were normalized to control cells (*n* = 6 experiments). (c, d) Control HEK293T cells were transduced with AAV encoding the mCherry reporter (no TMEM16F). The mCherry‐TMEM16F stably‐expressing cells and the mCherry control cells were co‐transduced with lentiviruses encoding α‐synA53T‐HA. The levels of α‐synA53T in the intracellular (cell lysate) and extracellular (cell media) fractions were analyzed. Results are normalized to WT TMEM16F cells. *n* = 3 experiments, 2‐tailed *t*‐test *p < 0.05, **p < 0.01. (e) Images of the morphology of the stable cell lines. Scale bar 50 μm. (f) Extracellular vesicles were purified from cell media of stably‐expressing cells (control cells: MCherry + α‐synA53T‐HA, WT cells: MCherry‐TMEM16F + α‐synA53T‐HA, and mutant cells: MCherry‐TMEM16F A703S + α‐synA53T‐HA). The vesicles were analyzed for various membrane protein markers and for α‐synA53T. Representative blots are presented (*n* = 3 experiments).

Since extracellular secretion of pathologic α‐syn from donor cells is a critical stage in the transfer to recipient cells, we examined whether the TMEM16F A703S regulates α‐syn extracellular secretion. To this end, HEK293T cells were transduced with lentiviruses (LV) expressing wild type (WT) or the A703S mutant TMEM16F attached to mCherry reporter (Figure 4a). Both WT and mutant lines express similar levels of TMEM16F as detected by flow cytometry (Figure 4b). Imaging of the cells revealed a comparable plasma membrane distribution of the WT and mutant TMEM16F (Figure 4a). However, analysis of recombinant Annexin V binding revealed an increase in cell‐surface PS exposure upon ionomycin treatment in the mutant cells (Figure 4b), indicating that the A703S mutant form has higher levels of phospholipid scramblase activity than the WT cells.

In order to examine this effect on α‐synA53T secretion, we positively selected TMEM16F‐expressing lines with mCherry and further transduced with LV encoding α‐synA53T‐HA. Then, we monitored the levels of α‐synA53T in the cultured media over several days (Figure 4c). The results indicated that the levels of extracellular α‐synA53T in the media are higher for the A703S mutant TMEM16F‐expressing cells than WT TMEM16F‐expressing cells, although both lines exhibit similar levels of intracellular α‐synA53T (Figure 4d). These results were validated by the comparison to mCherry control (no TMEM16F expression). Stable mCherry control cells were established using AAV transduction and selected according to mCherry reporter expression, and then transduced with LV encoding α‐synA53T‐HA. The different TMEM16F cell lines co‐expressing α‐synA53T are viable with no apparent morphological differences or cell death (Figure 4e), but there was a decrease in the levels of extracellular α‐synA53T in the control cells when compared to WT or mutant TMEM16F‐expressing cells (Figure 4d).

To investigate whether extracellular vesicles (EVs) are involved in TMEM16F‐regulated α‐syn secretion, we further isolated EVs from conditioned media of the cells with perturbations in TMEM16F (Figure 4c). We utilized membrane affinity spin columns, which were developed and optimized to recover the entire spectrum of EVs confirmed by markers, such as CD9 and CD63 (Enderle et al., 2015). Biochemical analysis of the vesicles showed no detection of calnexin to rule out microsomal contamination. In addition, GM130 was not detected in vesicle samples, suggesting the samples were free from contamination with cellular proteins (Figure 4f). We found increased α‐synA53T levels in the EVs of WT or A703S mutant TMEM16F‐expressing cells compared to control cells (Figure 4f). EVs derived from the A703S mutant TMEM16F cells showed an increase in α‐synA53T levels to some extent (Figure 4f). Notably, we could also detect mCherry‐TMEM16F itself in these vesicles.

**FIGURE 5:**
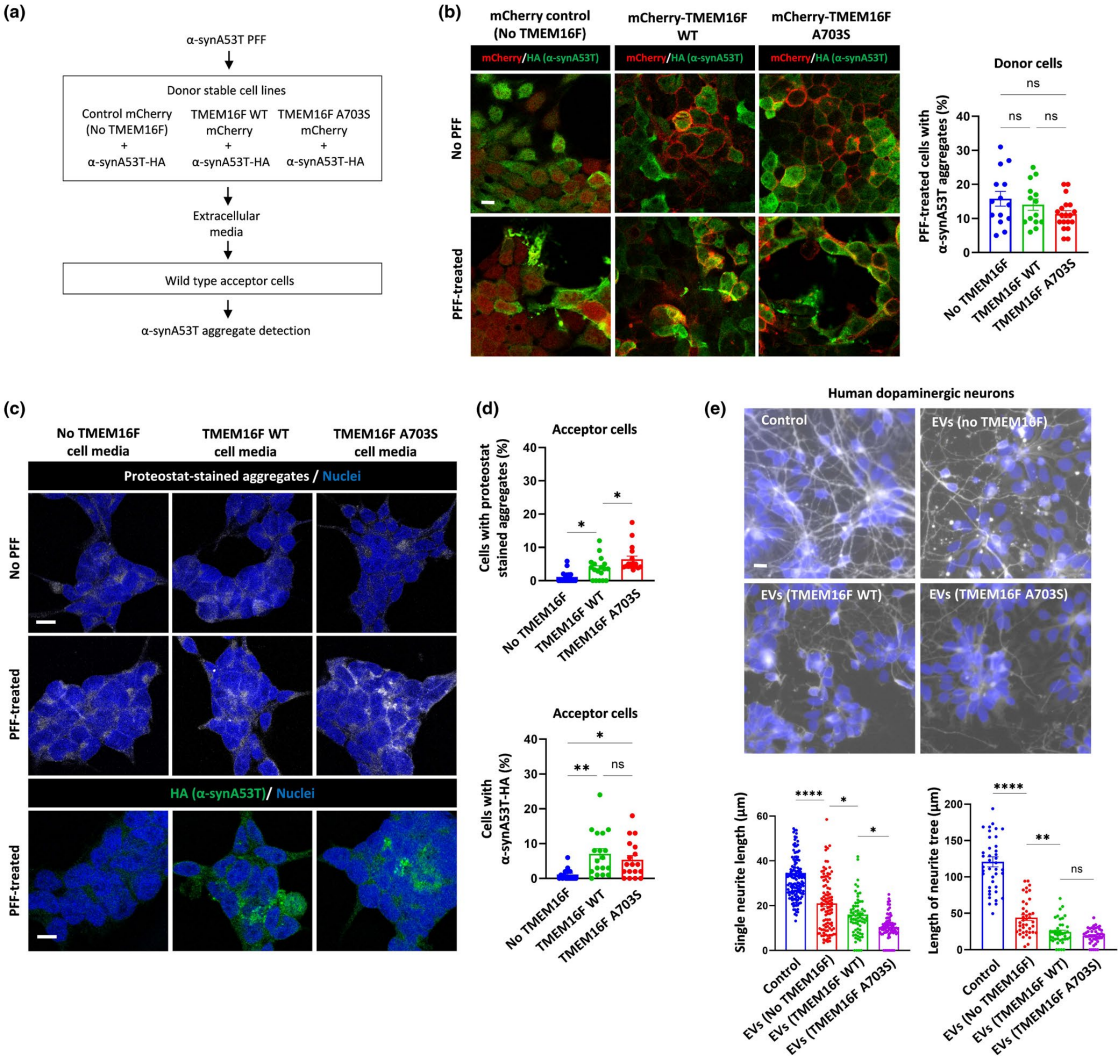
PFF modeling of TMEM16F‐regulated spread of α‐synucleinA53T in human cell lines and iPSC‐derived dopaminergic neurons. (a) Schematic representation of experimental conditions analyzed in b–d. Different stably expressing donor HEK293T cells (control cells: MCherry + α‐synA53T‐HA, WT cells: MCherry‐TMEM16F + α‐synA53T‐HA, and mutant cells: MCherry‐TMEM16F A703S + α‐synA53T‐HA) were incubated with buffer or recombinant α‐synA53T PFF (1 μg/mL). After 4 days, the number of cells with aggregates was analyzed by quantifying aggregation puncta of α‐synA53T (HA staining). (b) Images of the donor cells (mCherry in red and HA staining in green) after 4 days with or without PFF treatment. Scale bar 10 μm. The quantification of percentage of cells with aggregation puncta is presented in different image fields in *n* = 3 experiments (900–1800 cells were analyzed for each stable cell line). (c, d) Wild type HEK293T acceptor cells were incubated for 2 days with conditioned media from the PFF‐treated donor cell lines or with media from buffer‐treated donor cell lines (no PFF), and were analyzed for α‐synA53T transfer. (c) Images of acceptors cells stained for α‐synA53T‐HA (HA staining colored green) or probed with proteostat dye (colored gray) together with nuclei staining (colored blue). Scale bar 10 μm. (d) The quantification of percentage of cells with proteostat‐stained aggregates or positive to HA staining is presented in different image fields in *n* = 3 experiments (450–900 cells were analyzed for each condition). (e) Human iPSC‐derived dopaminergic neurons were incubated with extracellular vesicles (EVs) purified from conditioned media of the PFF‐treated donor cell lines. After 1 week, the neurons were stained for β‐tubulin III (DAPI used for nuclei staining), and analyzed for mean single neurite length and the mean total length of the complete neurite tree per neuron (50 neurons were analyzed for each condition). Image scale bar 10 μm. The results are from two independent iPSC differentiations. One‐way ANOVA (b, d, e). ns, nonsignificant, *p < 0.05, **p < 0.01, ****p < 0.0001.

We next explored effects of the A703S mutant TMEM16F on the spread of α‐synA53T between donor and acceptor HEK293T cells (Figure 5a–c). For this purpose, we utilized the α‐syn preformed fibrils (PFF) model in which initial PFF α‐syn seeds recruit cellular soluble monomers, in a homotypic seeding manner, to induce the formation of aggregates (Sanders et al., 2014). Donor cells were generated by the delivery of recombinant α‐synA53T PFF to different TMEM16F stable cell lines co‐expressing α‐synA53T (No TMEM16F control, TMEM16F WT, TMEM16F A703S). After several days, between 10% and 20% of the cells exhibited aggregated structures of α‐synA53T, compatible with previous studies (Sanders et al., 2014; Woerman et al., 2015). Conditioned media samples were next transferred from the different PFF‐treated donor cell lines to wild type cells (termed acceptor cells) to analyze cell‐to‐cell transfer of α‐synA53T. Interestingly, while the donor cell lines showed comparable levels of intracellular α‐synA53T expression and aggregation (Figure 5a,b), there were significant differences in α‐synA53T transfer to acceptor cells (Figure 5c,d). Acceptor cells incubated with WT or mutant TMEM16F‐derived conditioned media exhibited increased α‐synA53T immunostaining compared to control media‐incubated cells (Figure 5c,d). To assess whether TMEM16F‐mediated α‐synA53T transfer involves aggregated species, we stained the acceptor cells with proteostat dye enabling the detection of amyloid‐like aggregates. We found significant increase in cells with proteostat‐stained aggregates when incubated with mutant TMEM16F‐derived conditioned media compared to cells incubated with conditioned media derived from WT TMEM16F (Figure 5c,d).

Finally, we examined the possible toxic effects of donor cell‐derived conditioned media on vulnerable PD neurons (Figure 5e). EVs were isolated from conditioned media samples of the α‐synA53T PFF‐treated donor cell lines: EVs (No TMEM16F), EVs (TMEM16F WT), and EVs (TMEM16F A703S). The EVs were incubated with human induced pluripotent stem cell (iPSC)‐derived dopaminergic neurons we have previously characterized (Tripathi et al., 2024). Neurite degeneration was calculated by measuring the mean single neurite length and the mean total length of the complete neurite tree per neuron (Koch et al., 2015). We found that dopaminergic neurons treated with the EVs have decreased length of single neurite and the complete neurite tree. Compatible with our model, dopaminergic neurons treated with EVs (TMEM16F WT) and EVs (TMEM16F A703S) had a shorter single neurite length compared to the treatment with EVs (No TMEM16F) with a further neurite shortening caused by treatment with the EVs (TMEM16F A703S) (Figure 5e).

### 3.6 TMEM16F A703S structural model suggests a crosstalk between calcium coordination and scrambling activity

**FIGURE 6:**
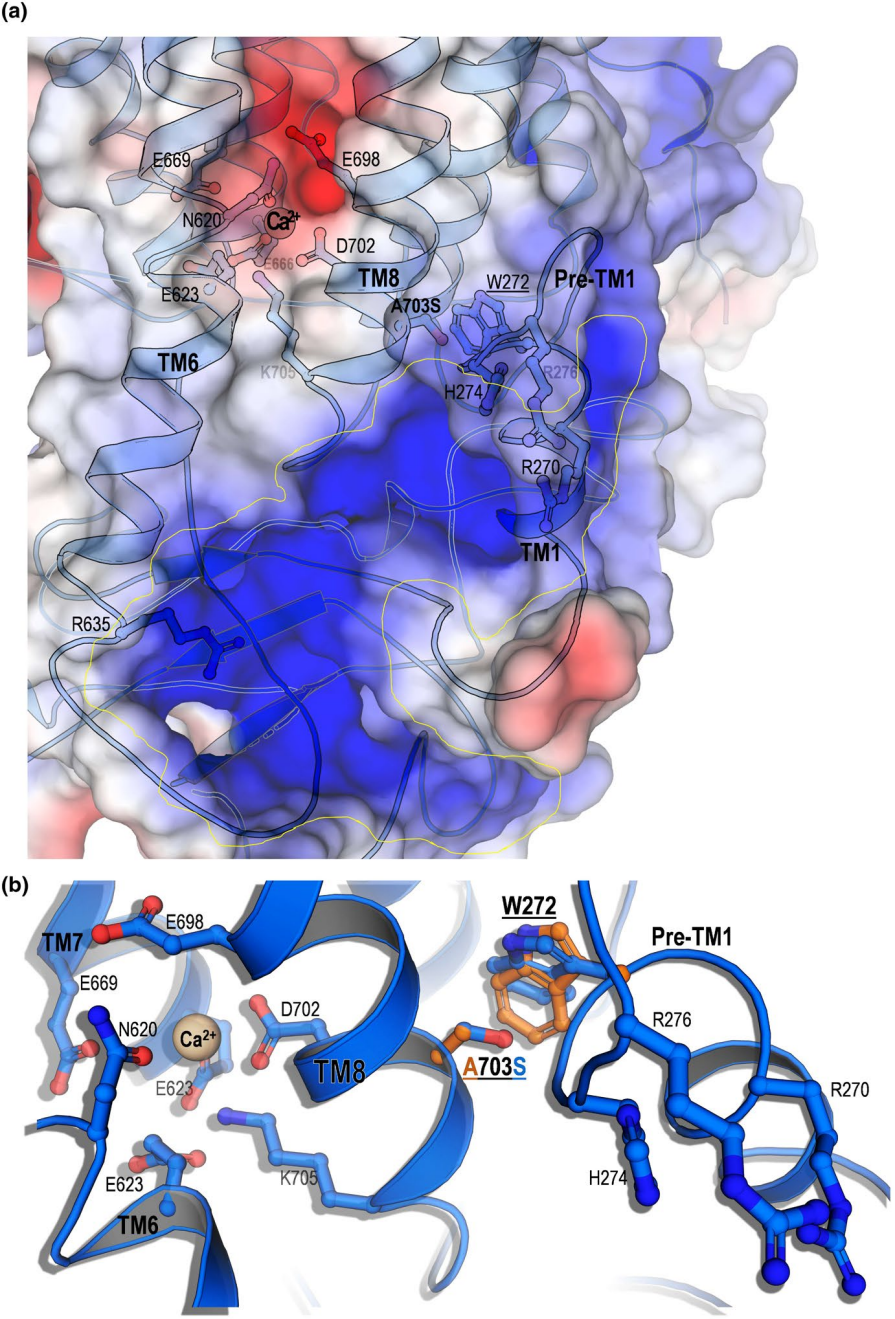
TMEM16F A703S structural model correlates calcium coordination with scrambling activity. (a) A structural model of the human TMEM16F containing the A703S was generated using AlphaFold2 and Phyre2. The structures of the mouse TMEM16F with Ca2+ (PDB 6P46) served as templates to model the Ca^2+^ binding residues within transmembrane 6 (TM6), TM7, and TM8. The positive patch involved in lipid association (yellow circled) comprising R635 located at the carboxy terminus of TM6, and R270, H274, and R276 located at the pre‐TM1 elbow. (b) A703 is located close to the negatively charged Ca^2+^ binding pocket and interacts with W272 within the pre‐TM1 elbow. The substitution of alanine 703 with serine pushes W272 towards the positive patch.

We sought to obtain structural insight into the A703S mutation. Using AlphaFold2 (Jumper et al., 2021) and Phyre2 (Kelley et al., 2015), we constructed models of the human WT and A703S TMEM16F. The structures of mouse TMEM16F with Ca^2+^ served as templates to model the Ca^2+^ binding residues (Feng et al., 2019). The mouse and the human TMEM16F proteins shared 90% sequence identity. The AlphaFold averaged predicted local distance difference test (pLDDT) scores of the obtained model indicate a very high confidence (Figure S6). Structurally, TMEM16F and TMEM16A are highly similar; however, a Ca^2+^ ion was found to bind and regulate the scramblase activity of TMEM16F. This Ca^2+^ is coordinated by N620 and E623 on transmembrane 6 (TM6), E669 on TM7 and E698 and D702 on TM8 (Figure 6a). Moreover, R635 located at the carboxy terminus of TM6, and R270, H274, and R276 located at the pre‐TM1 elbow form a positive patch that probably interacts with the lipid head groups to induce membrane curvature and distortion. This increases the cell surface exposure of PS and facilitates the release of extracellular vesicles (Feng et al., 2019). The molecular details of how Ca^2+^ regulates scramblase activity are yet to be deciphered. However, it has been reported that alanine substitution of mouse TMEM16F corresponding to human residues R270, H274, and R276 (triple mutant) increases Ca^2+^ influx, PS exposure, and the generation of plasma membrane vesicles (Feng et al., 2019). We found that A703S mutation also increases cell surface PS exposure but this perturbation could be mild and may not be sufficient to induce substantial elevation of extracellular vesicles. Intriguingly, A703 is located at a pivotal point on TM8. D702 coordinates Ca^2+^ binding while A703 forms interaction with W272 at the pre‐TM1 elbow (Figure 6b). Therefore, we suggest that this TM8 functions as a pillar that transduces the information of Ca^2+^ binding/occupancy to the basic cluster of R270, H274, and R276, on the pre‐TM1 elbow. Our model proposes that the substitution of alanine 703 with serine increases the proximity of W272 towards the positive patch, and thus mimics to some extent Ca^2+^ coordination in a constitutive manner.

## 4 DISCUSSION

Our study identifies TMEM16F/ANO6 as a regulator of pathologic α‐syn extracellular secretion and spread in neurons and in the brain. Less α‐synA53T cell‐to‐cell spread was detected in TMEM16F‐depleted neurons and mouse brains with no detectable effects on the turnover of α‐synA53T via autophagy and the ubiquitin‐proteasome system. In this context, we identified the FDA‐approved drug Niclosamide as a potential modulator of α‐syn spread. Niclosamide, which inhibits the TMEM16 family was originally approved for treating tapeworm infections but also displays a broad anticancer and antiviral activities and is considered a candidate for drug repurposing (Braga et al., 2021; Cabrita et al., 2019; Xu et al., 2016). Surprisingly, our results indicate that Niclosamide exacerbates pathologic α‐syn spread in neurons. It could be that this drug targets multiple members of the TMEM16 family (TMEM16F and TMEM16A) and is also involved in TMEM16F‐independent signaling pathways (Arend et al., 2016). Several modulators of TMEM16 family members target both TMEM16A and TMEM16F. Some of these molecules were identified by screening for anion channel blockers, regulators of calcium oscillations, and syncytia fusion formation (Braga et al., 2021; Cheng et al., 2023; Feng et al., 2019; Lam et al., 2022). Since TMEM16F, but not TMEM16A, is also a lipid scramblase (Pedemonte & Galietta, 2014; Suzuki et al., 2013), future screens focusing on modulators of calcium‐induced lipid scrambling may yield specific TMEM16F inhibitors.

While exogenously prepared α‐syn pre‐formed fibrils have significantly promoted the understanding of propagation and cell‐to‐cell spread (Kim et al., 2019; Lee et al., 2014; Luk et al., 2012; Sacino et al., 2014) they cannot fully recapitulate physiologically secreted α‐syn. By distinguishing between α‐syn donor and receiving neurons, our modulated AAV system can address protein secretion mechanisms that may contribute to the early initiation and progress of α‐syn spread. How do changes in pathologic α‐syn secretion and spread affect PD clinical phenotypes? Considering Braak's hypothesis that α‐syn pathology in non‐dopaminergic structures predates the onset of motor symptoms in PD, and then spreads throughout the brain (Braak et al., 2004), it is plausible that inhibition of pathologic α‐syn secretion or uptake will be clinically beneficial. Indeed, recent studies identified the membrane proteins, LAG3 and LRP1 as regulators of α‐syn uptake from the extracellular space with implications for α‐syn spread and pathology in mouse brains (Chen et al., 2022; Mao et al., 2016). Moreover, since the rate of spread is probably dependent on pathways of extracellular secretion of α‐syn from donor neurons and subsequent uptake by recipient cells, focusing on these pathways could facilitate identification of potential disease modalities.

The abnormally active TMEM16F/ANO6‐p.Ala703Ser variant we identified, which appears unique to the AJ population, enhances lipid scramblase activity and pathologic α‐syn secretion in cells. Our structural modelling of the TMEM16F Ala703Ser substitution highlights a role for TM8 of TMEM16F in Ca^2+^ coordination. However, the model cannot exclude the possibility that the mutation stabilizes Ca^2+^ binding. In this study, we have also attempted to provide insights on how Ca^2+^‐induced scramblase activity correlates with pathologic α‐syn secretion. Non‐classic secretory pathways have been suggested to be involved in the release of α‐syn from cells, including secretion of non‐vesicular soluble α‐syn (Wu et al., 2023) or α‐syn‐associated with exosomes and other extracellular vesicles (Alvarez‐Erviti et al., 2011; Danzer et al., 2012; Stuendl et al., 2021). Exosomes are secreted by multivesicular bodies (Subra et al., 2010), while other extracellular vesicles are budding from the plasma membrane. Deficient TMEM16F scramblase activity in mouse and Scott syndrome patient‐derived platelets is correlated with decreased microvesicle release (Fujii et al., 2015). In other cell types, such as thymocytes, TMEM16F was shown to mediate secretion of extracellular vesicles during plasma membrane repair after pore formation (Wu et al., 2020). TMEM16F is also involved in the formation of multivesicular bodies (Hu et al., 2016), and it is plausible that it is integrated in vesicles, like other proteins in multivesicular bodies or plasma membrane‐residing proteins. Since pathologic α‐syn can increase intracellular Ca^2+^ and is involved in membrane pore formation (Danzer et al., 2007; Fusco et al., 2017; Tsigelny et al., 2012), it may activate TMEM16F‐mediated secretion of extracellular vesicles. Moreover, several α‐syn forms with pathologic functions, such as the A53T α‐syn we have utilized in our experiments, tend to be associated with vesicular secretion (Gustafsson et al., 2018). Nevertheless, the involvement of TMEM16F in other forms of unconventional secretion of α‐syn remains to be elucidated.

Our clinical analysis of the AJ cohort of PD patients carrying the TMEM16F p.Ala703Ser variant has some limitations (Supplementary results; Appendix S1 and Table S3). We did not have an available assessment of α‐syn pathology in the patients analyzed to test whether the TMEM16F variant modifies age‐at‐onset of motor symptoms. In addition, other clinical parameters, such as prodromal symptoms and cognitive functions were lacking in this patient cohort. Therefore, we were unable to verify whether the TMEM16F p.Ala703Ser variant modifies PD. However, at the cellular level, the detection of TMEM16F in secreted vesicles may hint at its presence in the plasma and cerebrospinal fluid of patients. Importantly, our study provides insights into the mechanisms of α‐syn spread and highlights TMEM16F as a potential therapeutic target in synucleinopathies.

## AUTHOR CONTRIBUTIONS

S.C.A. and F.A.S. performed and analyzed neuronal and cell‐based experiments; D.F.M., E.B., and S.C.A. performed and analyzed in vivo experiments; Y.B. performed molecular biology experiments and cloning; U.T., W.A.R., and S.S. performed iPSC experiment and differentiation; G.P. performed structural modeling; T.G. and N.G. recruited and diagnosed patients; A.O.U., O.G., and M.G.W. performed and analyzed genetics experiments; A.A. supervised the study and obtained funding; All authors were involved in writing the paper.

## FUNDING INFORMATION

We are grateful for funding from The Aufzien Family Center for the Prevention and Treatment of Parkinson's Disease (grant to A.A, scholarship to S.C.A), Marguerite Stolz Research Fellowship Fund (grant to A.A), Yoran Institute for Human Genome Research (scholarship to F.A.S).

## CONFLICT OF INTEREST STATEMENT

The authors report no competing interests.

## Supporting information


Appendix S1.

## Data Availability

This study includes no data deposited in external repositories. Software used for statistical analysis, and whole‐genome‐sequencing analysis are listed in the Methods section.
