# Supplementary material for: TMEM16F regulates pathologic α‐synuclein secretion and spread in cellular and mouse models of Parkinson's disease
Source: Aging Cell. 2024 Nov 2;24(2):e14387. doi: 10.1111/acel.14387 (PMC11822650; doi:10.1111/acel.14387)
Supplement: Supplementary file 1 — Appendix S1. [file ACEL-24-e14387-s001.zip › Cohen Adiv et al. Supplementary information.pdf]

<u>Supplementary information (Cohen-Adiv et al.)</u>

The supplementary information section incudes supplementary results, Supplementary Table 1 (supplied as a separate xls. file), Supplementary Table 2, Supplementary Table 3, and Supplementary Figure 1-6.

Supplementary results

<u>Characterization of *TMEM16F*- p.Ala703Ser in the AJ patient cohort</u>

The frequency of *TMEM16F*- p.Ala703Ser carriers observed in the 1200 AJ-PDs is not statistically different from the frequency in gnomAD-AJ-non-neuro cases (1.83% carrier rate in our population compared to 1.68% in controls, allelic odds ratio (OR) = 1.09, 95% CI: 0.68-1.76, *p* = 0.7136). As age-at-onset (AAO) of motor symptoms is one of the markers for the severity of PD, we evaluated whether *TMEM16F*- p.Ala703Ser modifies AAO in the group of AJ PD patients that do not carry mutation in the GBA and/or LRRK2 genes (*n*=785, PD- non-carrier (NC) with AAO information). The PD-NC average AAO in those harboring *TMEM16F*- p.Ala703Ser (66.7 years, *n*=16) was 5.18 years later than in those without the SNV (61.5 years, *n*=769; *p*= 0.063, t-test) but without statistical significance, even after correcting for sex (linear regression analysis with sex and genotypes as covariates, excluding outliers, beta (geno)= 4.8, *p*= 0.066).

Supplementary Table 2. *TMEM16F/ANO6* coding variants identified by whole-genome-sequencing in 250 Ashkenazi-Jewish PD/DLB patients.

Location (hg38)	Ref	Alt	rs ID	Annotation	Effect	Phred CADD score	# of carriers	gnomAD-v2.1.1 Allele Frequency in AJ-non-neuro cases [a]	gnomAD-v2.1.1 Allele Frequency in non-AJ-non-neuro cases [a]
12:45292920	T	A	rs12822001	NP_001136150.1: p.Met1Lys; NP_001397902.1: p.Met1Lys	Loss of initiator coding variant (LoF)	13.76	1	0.001208	0.001392
12:45348064	G	A	rs2162321	NP_001020527.2: p.Ala128Thr *	Missense	23.50	3	0.008364	0.012108
12:45401867	A	G	rs200331398	NP_001020527.2: p.Lys487Glu	Missense	11.88	1	0.00	0.000010
12:45416794	G	T	rs202121654	NP_001020527.2: p.Ala703Ser	Missense	24.30	2	0.008209	0.0000539
12:45429234	A	G	rs59243955	NP_001020527.2: p.Met886Val *	Missense	16.08	3	0.008060	0.002610
12:45429264	C	T	rs758435453	NP_001020527.2: p.Arg896Trp	Missense	20.10	1	0.001771	0.000337
12:45439774	G	A	rs142698881	NP_001136151.1: p.Asp876Asn	Missense	0.02	1	0.003629	0.004458
12:45439811	-	TAA	rs774393824	NP_001136151.1: p.Phe888delinsLeuIle	Missense	23.60	5	0.006449	0.000249

*Linked variants. [a] individuals who were not ascertained for having a neurological condition in a neurological case/control study.

AJ=Ashkenazi Jews.

Supplementary Table 3. Characteristics of 1200 unrelated, consecutively recruited, Parkinson's disease patients of Ashkenazi Jewish origin.

Genotype	Number of PD patients (%)	Number of females (%)	Average age at onset (±SD)
Carriers of *GBA* mutations [a]	235 (20%)	94 (40%)	58.7 (10.5)
Carriers of *LRRK2*-G2019S mutation	145 (12.1%)	65 (44.8%)	58.4 (10.5)
Carriers of dual mutations	25 (2.1%)	17 (68%)	58.5 (9.9)
Carriers of *SMPD1*-L302P mutation	8 (0.7%)	3 (37.5%)	55.5 (12.7)
Non-Carrier (NC)	787 (65.6%)	297 (37.7%)	61.6 (11.0)
Total	1200	476	60.5 (11.2)

[a]- 10 *GBA* mutations (severe *GBA* mutations=c.84insG, IVS2+1G>A, p.V394L, p.L444P; mild *GBA* mutations= p.R496H, p.N370S, 370Rec; risk alleles=p.E326K, p.T369M; and *GBA*-p.R44C. SD=standard deviation.

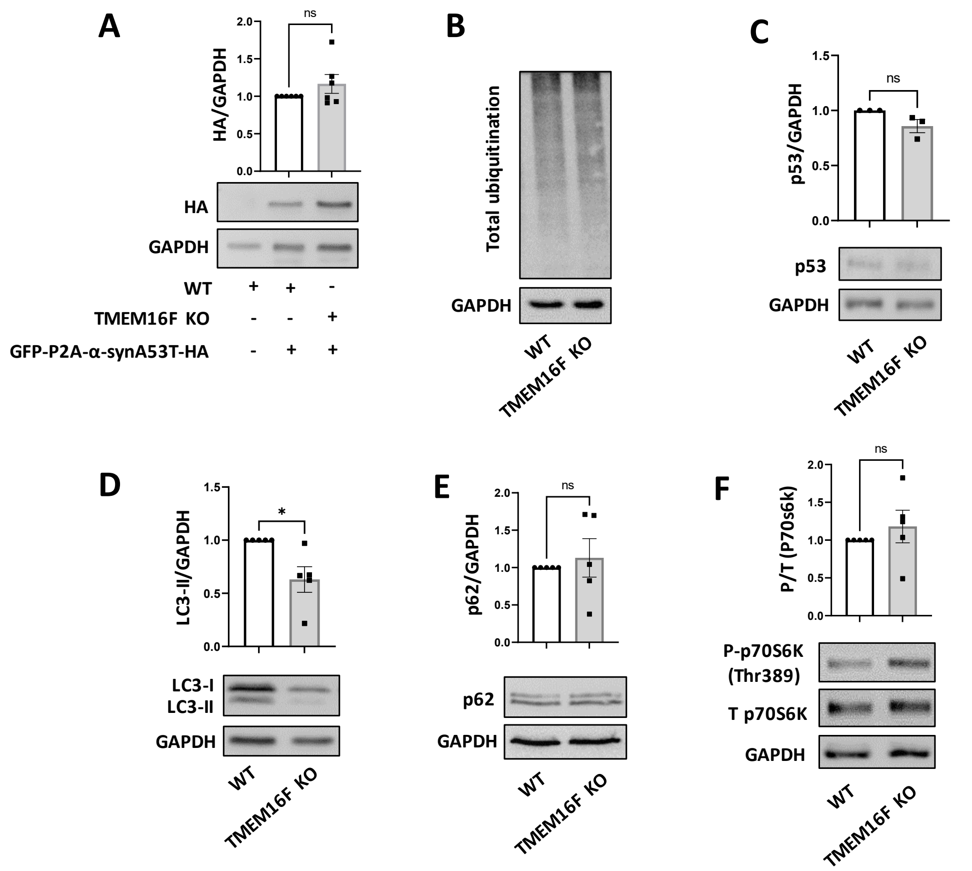

Supplementary Figure 1. Analysis of ubiquitin-proteasome and autophagy pathways in TMEM16F KO neurons. WT and TMEM16F KO neurons were transduced with the AAV encoding eGFP-P2A-α-syn A53T-HA and cell lysates were analyzed for the levels of: (A) α-syn A53T-HA. (B) polyubiquitinated proteins. (C) p53 as proteasome substrate. (D) LC3-II. (E) p62 as autophagy substrate. (F) Phospho-p70 S6 Kinase (Thr389) as mTORC1substrate. Results are normalized to control WT neurons. n= 3-6 independent cultures. 2-tailed t-test. ns non-significant, * $p < 0.05$.

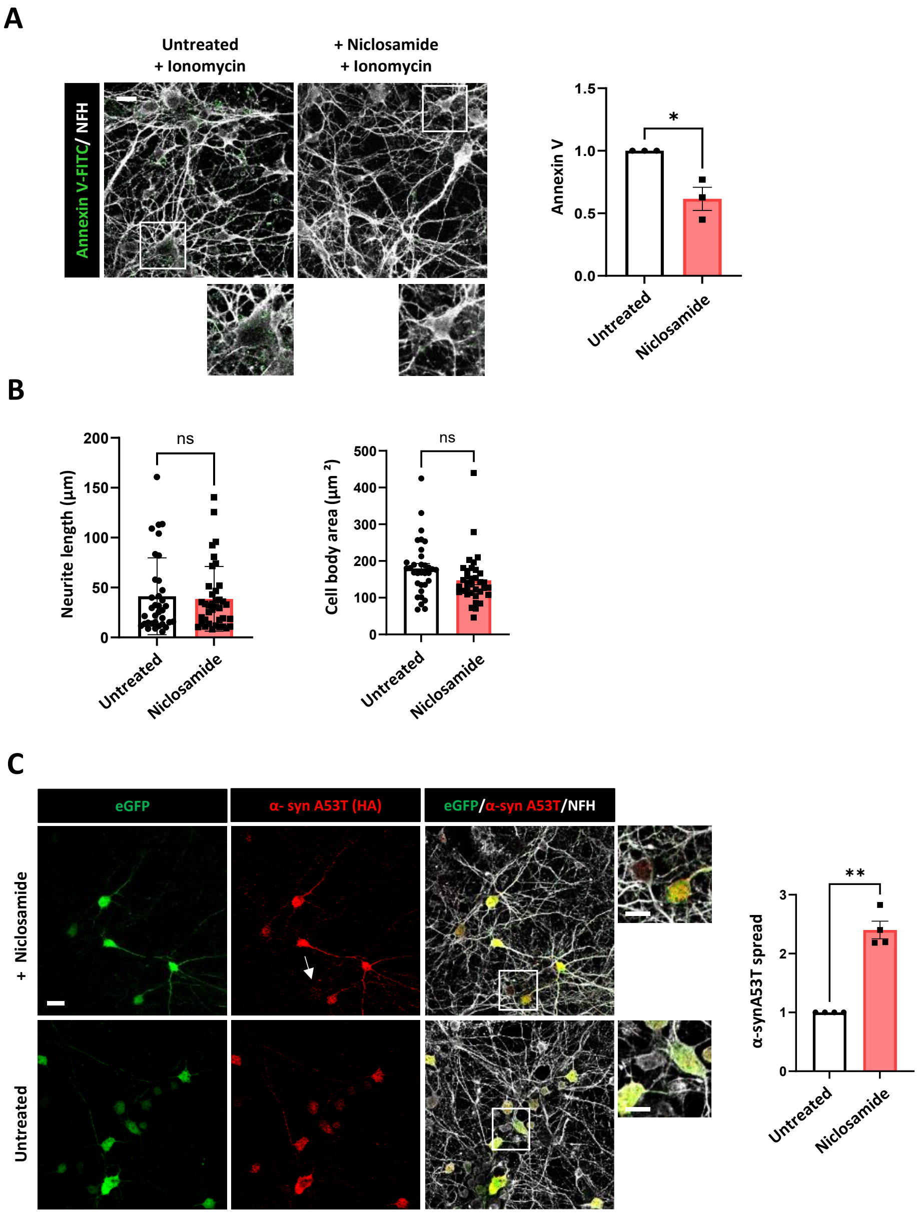

Supplementary Figure 2. Niclosamide aggravates neuronal α-syn A53T spread. (A) Mouse primary cortical neurons were treated with Niclosamide (100 nM) for 1 hour at 37°C. Extracellular PS exposure was detected by FITC-Annexin V binding in ionomycin-treated (250 nM for 10 min) Niclosamide-exposed neurons. Confocal images present FITC fluorescence (colored green) and neuronal morphology marker NFH (colored gray). Scale bar 20 µm. FITC intensity in different image fields was obtained and normalized to control untreated neurons. n=3 independent cultures. (B) Analysis of neurite length and cell body area in the NFH-stained control and Niclosamide-exposed neurons. Results are the average of the morphological parameters in different image fields

in n=3 independent cultures. (C) Primary neurons were transduced with AAV encoding eGFP-P2A-α-synA53T-HA following a treatment with two repeated doses of Niclosamide (100 nM x 2) for a week prior to spread analysis. NFH staining (colored gray), eGFP (colored green), α-syn A53T-HA (HA staining, colored red). Images of the transduced neurons are shown (scale bar 20 μm) for donor neurons (NFH^+/ $eGFP^+$/ α-syn A53T-HA^+) and recipient neurons (NFH^+/ $eGFP^-$/ α-syn A53T-HA^+, recipient neurons marked with arrows and showed in insets, inset scale bar 10 μm). Between 100 to 150 neuronal cell bodies were quantified per experiment. Results were normalized to control neurons. n=4 independent cultures. (A) 1-tailed t-test and (B,C) 2-tailed t-test. ns non-significant, * $p < 0.05$, ** $p < 0.01$.

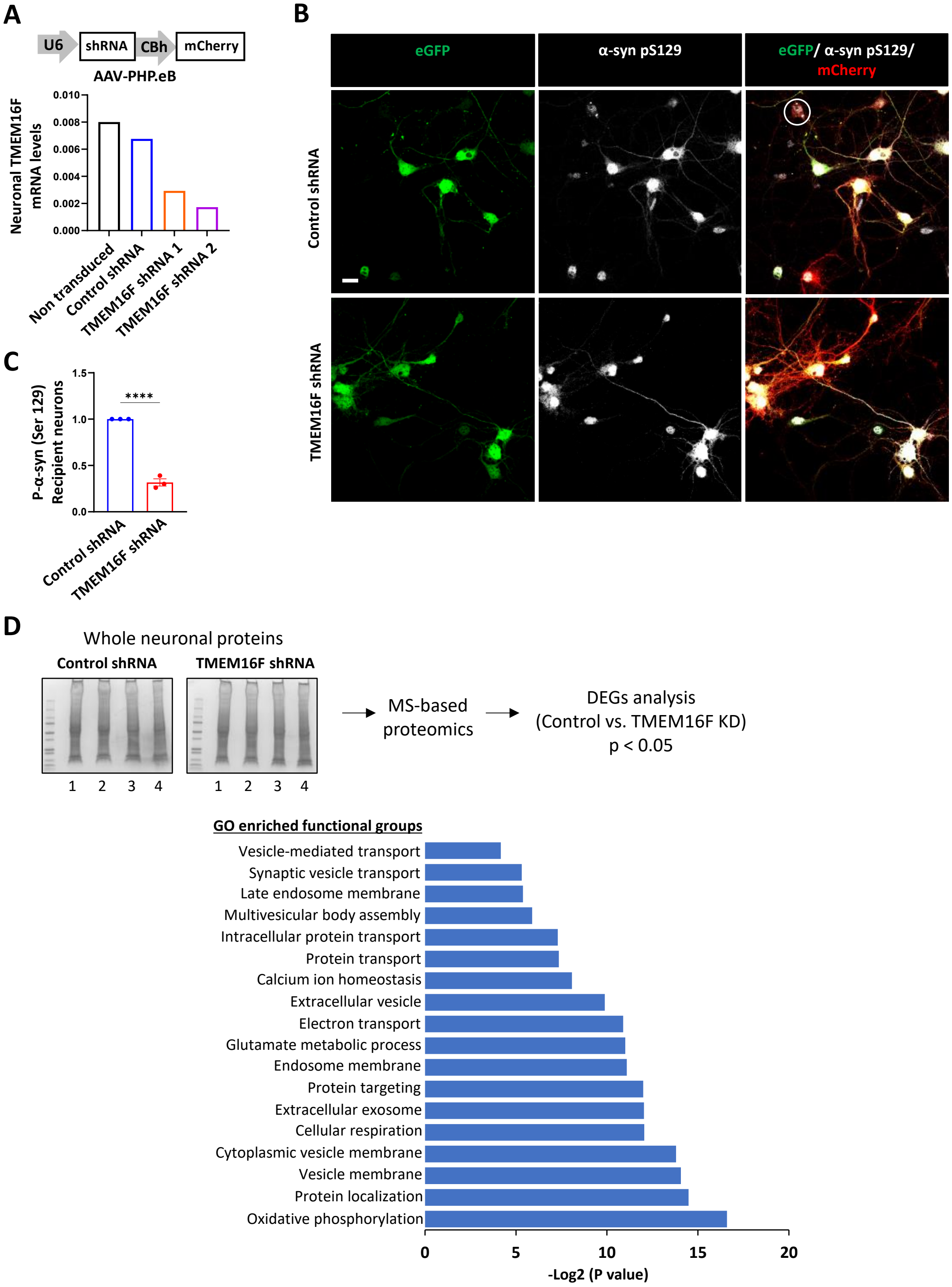
A
U6
shRNA
CBh
mCherry
AAV-PHP.eB
Neuronal TMEM16F mRNA levels
0.010
0.008
0.006
0.004
0.002
0.000
Non transduced
Control shRNA
TMEM16F shRNA 1
TMEM16F shRNA 2
B
eGFP
α-syn pS129
eGFP/ α-syn pS129/ mCherry
Control shRNA
TMEM16F shRNA
C
P-α-syn (Ser 129) Recipient neurons
1.5
1.0
0.5
0.0
****
Control shRNA
TMEM16F shRNA
D
Whole neuronal proteins
Control shRNA
TMEM16F shRNA
1 2 3 4
1 2 3 4
MS-based proteomics
DEGs analysis (Control vs. TMEM16F KD) p < 0.05
GO enriched functional groups
Vesicle-mediated transport
Synaptic vesicle transport
Late endosome membrane
Multivesicular body assembly
Intracellular protein transport
Protein transport
Calcium ion homeostasis
Extracellular vesicle
Electron transport
Glutamate metabolic process
Endosome membrane
Protein targeting
Extracellular exosome
Cellular respiration
Cytoplasmic vesicle membrane
Vesicle membrane
Protein localization
Oxidative phosphorylation
0
5
10
15
20
-Log2 (P value)

Supplementary Figure 3. Effects of neuronal TMEM16F knockdown on α-syn pathologic spread and cellular pathways. (A) Mouse primary cortical neurons were transduced with AAV encoding for control scrambled shRNA or for one of two different TMEM16F shRNAs (TMEM16F targeting sh1 or sh2) as well as encoding for mCherry reporter. TMEM16F knockdown (KD) levels were analyzed by qRT-PCR relative to GAPDH. (B, C) Control and TMEM16F KD neurons were transduced with AAV encoding eGFP-P2A-α-synA53T-HA. mCherry signal (colored red), eGFP (colored green), phosphorylated S129 α-syn (P-S129 α-syn, colored gray). (B) Images of the transduced neurons are shown for donor neurons (mCherry$^+$/ eGFP$^+$/ P-S129 α-syn$^+$) and recipient neurons (mCherry$^+$/ eGFP $^-$/ P-S129 α-syn$^+$), recipient neurons marked with circle. Scale bar 20 µm. (C) Between 60 to 130 neuronal cell bodies were quantified per experiment. The percentage of recipient neurons with P-S129 α-syn staining was calculated in control and TMEM16F KD neurons. Results were normalized to control neurons. n=3 independent experiments. 2-tailed t-test, **** $p < 0.0001$. (D) The global effect of TMEM16F KD on neuronal pathways was analyzed by proteomics analysis. Equivalent amount of whole protein samples were processed and analyzed by mass spectrometry. Differentially expressed genes (DEGs) with p-value cutoff $p < 0.05$ between the groups (control shRNA vs. TMEM16F KD, n=4) were identified. Selected gene ontology (GO) enriched functions (performed by DAVID) are presented as bar-graphs ranked by $-\log_2 P$ value.

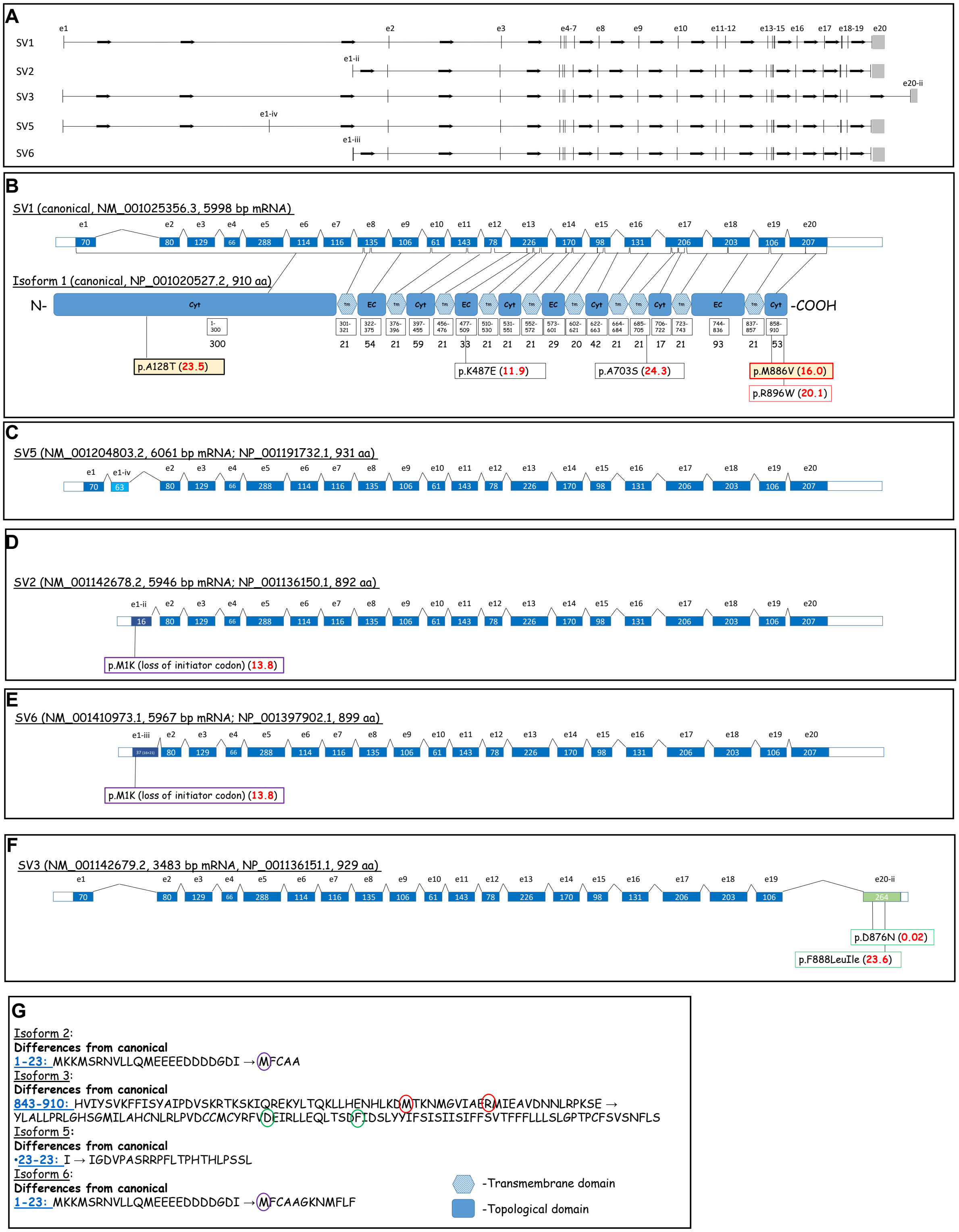
A
SV1
SV2
SV3
SV5
SV6
e1
e2
e3
e4-7
e8
e9
e10
e11-12
e13-15
e16
e17
e18-19
e20
e1-ii
e20-ii
e1-iv
e1-iii
B
SV1 (canonical, NM_001025356.3, 5998 bp mRNA)
Isoform 1 (canonical, NP_001020527.2, 910 aa)
N-
-COOH
p.A128T (23.5)
p.K487E (11.9)
p.A703S (24.3)
p.M886V (16.0)
p.R896W (20.1)
C
SV5 (NM_001204803.2, 6061 bp mRNA; NP_001191732.1, 931 aa)
D
SV2 (NM_001142678.2, 5946 bp mRNA; NP_001136150.1, 892 aa)
p.M1K (loss of initiator codon) (13.8)
E
SV6 (NM_001410973.1, 5967 bp mRNA; NP_001397902.1, 899 aa)
p.M1K (loss of initiator codon) (13.8)
F
SV3 (NM_001142679.2, 3483 bp mRNA, NP_001136151.1, 929 aa)
p.D876N (0.02)
p.F888LeuIle (23.6)
G
Isoform 2:
Differences from canonical
1-23: MKKMSRNVLLQMEEEEDDDDGDI → MFCAA
Isoform 3:
Differences from canonical
843-910: HVIYSVKFFISYAIPDVSKRTKSKIQREKYLTQKLLHENHLKDMTKNMGVIAERMIEAVDNNLRPKSE →
YLALLPRLGHSGMILAHCNLRLPVDCCMCYRFVDEIRLLEQLTSDFIDSLYYIFSISIISIFFSVTFFFLLLSLGPTPCFSVSNFLS
Isoform 5:
Differences from canonical
•23-23: I → IGDVPASRRPFLTPHTHLPSSL
Isoform 6:
Differences from canonical
1-23: MKKMSRNVLLQMEEEEDDDDGDI → MFCAAGKNMFLF
-Transmembrane domain
-Topological domain

Supplementary Figure 4. TMEM16F/ANO6 splice variants and isoforms. (A) Five TMEM16F splice variants (SV), drawn to scale. Vertical lines are exons. Shaded squares are 3' UTR. (B) TMEM16F canonical splice variant and isoform, drawn in details, but not to scale. Exons are marked in blue rectangles, numbered (e), and length (bp) are noted inside. Under the splice variant figure, the translation to a specific isoform is denoted. The exons corresponding to each translated domain are marked. At the protein level, the topological domains are represented by blue rectangles (either cytological =Cyt, or extra cellular= EC), while the transmembrane domains (tm) are marked with hexagons (UniProt, by curation or by similarity). Boxed numbers are the amino acid numbers, and the size of each domain is written under the box. Five TMEM16F single nucleotide variants (SNVs) that were observed in the AJ-PD cohort are also marked at the protein level. (C-F) TMEM16F additional splice variants drawn in details, but not to scale. The differences between the isoforms are marked with different colors. Three additional SNVs were observed. Of the Eight TMEM16F SNVs that were observed in the AJ-PD cohort, three (p.Ala128Thr, p.Lys487Glu, and p.Ala703Ser) are common to all TMEM16F isoforms. p.Met886Val and p.Arg896Trp, are shared among all isoforms except isoform 3. p.Met1Lys, is unique to isoforms 2 and 6, and p.Asp876Asn and p.Phe888delinsLeuIle, which are located at the C-terminus of the protein, are unique to isoform 3. Two SNVs, p.Ala128Thr and p.Met886Val, are linked. (G) The amino acids changes for each isoform compared to the canonical sequence. The SNVs are circled, with colors matched to specific splice variants.

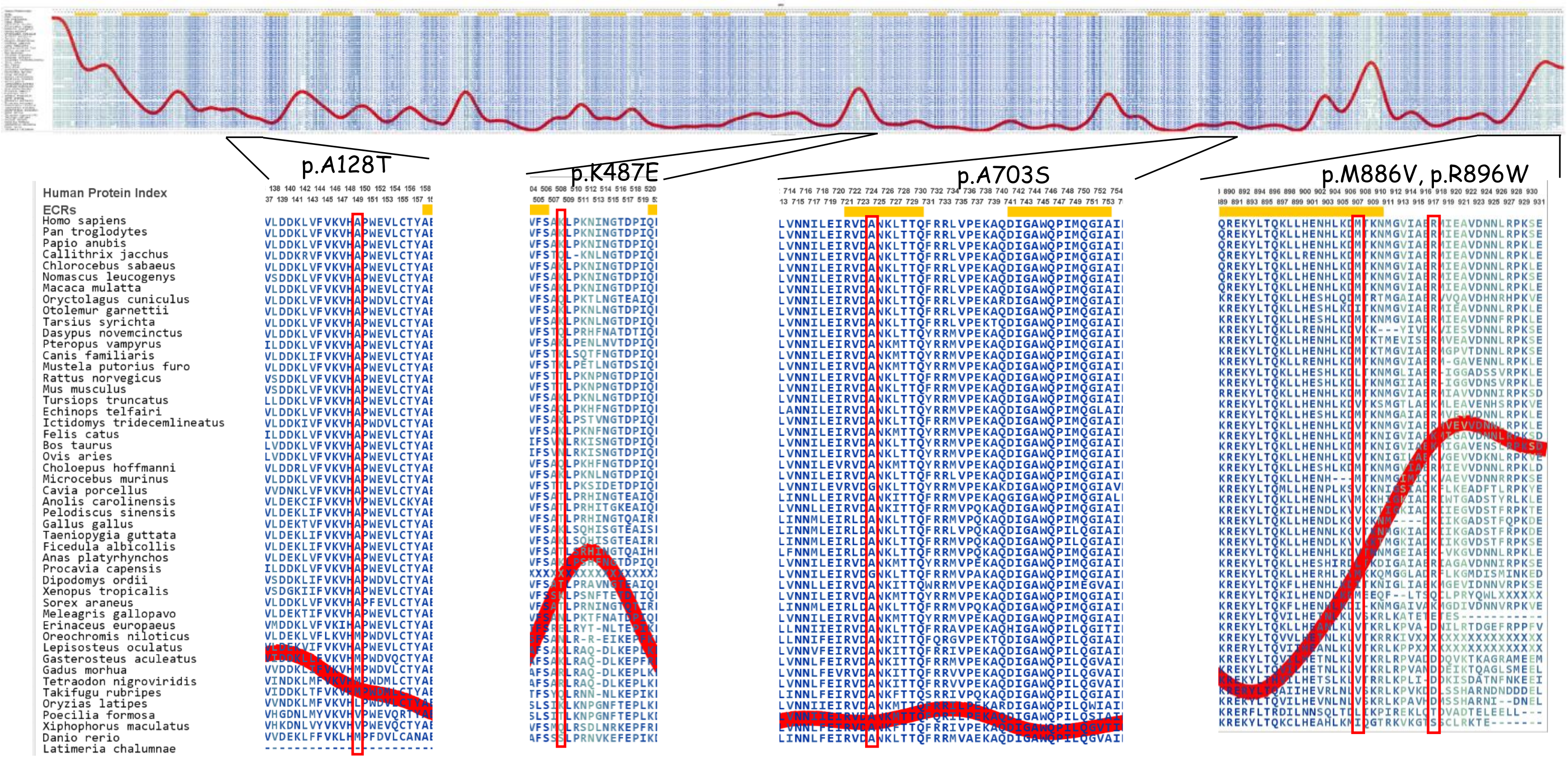

Supplementary Figure 5. TMEM16F evolutionary conservation (by Aminode). Multiple sequence alignments and evolutionary constrained regions (ECRs) of TMEM16F generated by Aminode. Upper panel presents the full TMEM16F protein, and the lower panel presents a higher magnification version of the five variants observed in the AJ-PDs with annotations to the canonical TMEM16F. The red line represents the relative rate of amino acid substitution calculated at each protein position. Local minima (highlighted by yellow bars above the graph) are ECRs with relatively low amino acids substitution rates. Peaks (local maxima) indicate regions with relatively high substitution rates.

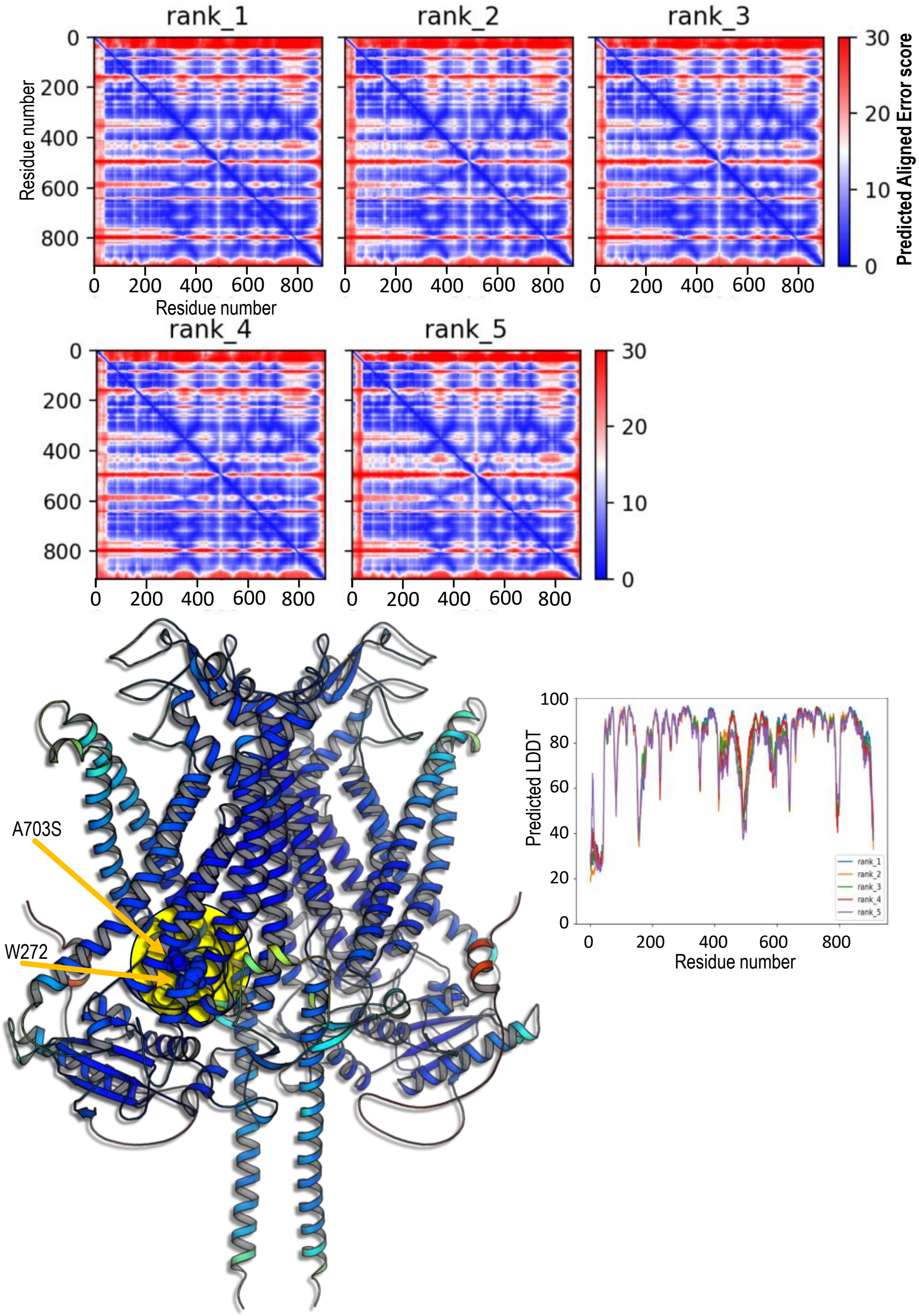

Supplementary Figure 6. Quality assessment of the alphafold models. Five highly similar alphafold models were obtained. Top plots show the Predicted Aligned Error (PAE) heat maps. The Bottom plot shows the pLDDT (predicted local distance difference test) confidence scores for the human TMEM16F A703S model.
